# Nonclassical MHC‐I Molecules: Emerging Therapeutic Targets in Next‐Generation Immunotherapy

**DOI:** 10.1002/mco2.70742

**Published:** 2026-04-21

**Authors:** Wanlin He, Andrew J. McMichael

**Affiliations:** ^1^ Center For Immuno‐Oncology Nuffield Department of Medicine University of Oxford Oxford UK; ^2^ State Key Laboratory of Oral Diseases & National Center For Stomatology and National Clinical Research Center For Oral Diseases West China Hospital of Stomatology Sichuan University Chengdu China; ^3^ Chinese Academy of Medical Sciences Oxford Institute Nuffield Department of Medicine University of Oxford Oxford UK

**Keywords:** antigen presentation, immunotherapy, immune modulation, innate–adaptive crosstalk, nonclassical MHC‐I molecules

## Abstract

Immunotherapies have transformed the treatment of cancers and infectious diseases by harnessing the precision and adaptability of the immune system. Central to these advances is the major histocompatibility complex (MHC) system, with classical MHC‐I molecules well documented for their role in immune surveillance. MHC‐dependent therapies, including immune checkpoint blockade (ICB), T cell receptor (TCR)‐engineered therapies, and cancer vaccines, have shown substantial clinical promise. However, their broader efficacy is hindered by the extreme polymorphism of classical MHC‐I molecules, susceptibility to immune evasion, and frequent downregulation in many disease settings. In contrast, nonclassical MHC‐I molecules, including HLA‐E, HLA‐F, HLA‐G, CD1, and MR1, offer alternative therapeutic opportunities. Shaped by strong evolutionary conservation, these molecules exhibit limited polymorphism, specialized antigen repertoires, distinct trafficking behaviors, and the capability to engage both innate and adaptive immune cells. In this review, we synthesize current knowledge of the structural biology, antigen presentation pathways, receptor interactions, and immunoregulatory functions of nonclassical MHC‐I molecules. We further highlight emerging therapeutic strategies, including immune checkpoint modulation, cargo‐based ligands, conformation‐specific biologics, vaccines, and cellular therapies, while critically evaluating translational challenges. By linking specialized structural and functional features to therapeutic design, this review provides a unified framework for exploiting nonclassical MHC‐I molecules as next‐generation targets in immunotherapy.

## Graphical abstract

Immunotherapies dependent on classical MHC‐I molecules face significant challenges, including extreme polymorphism and frequent downregulation in pathological conditions. This review discusses how nonclassical MHC‐I molecules (HLA‐E, HLA‐F, HLA‐G, CD1, MR1) may potentially circumvent these limitations through restricted genetic diversity, stable expression, and the capacity to present diverse antigens. Their distinctive ability to engage both innate and adaptive immune cells positions them as versatile and promising targets for universal, next‐generation immunotherapies against cancers and infectious diseases.

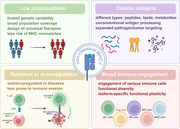

## 1 Introduction

The major histocompatibility complex (MHC), known as human leukocyte antigens (HLA) in humans, is one of the most evolutionarily conserved and functionally essential elements of the vertebrate immune system [1, 2]. MHC Class I (MHC‐I) molecules are ubiquitously expressed on nucleated cells and play a central role in immune surveillance. Their primary function is to present endogenous peptides, including those derived from intracellular pathogens and tumor‐associated proteins, to CD8+ cytotoxic T lymphocytes (CTLs), thereby enabling the immune system to recognize and eliminate infected or malignant cells [3]. MHC‐I molecules are fundamental to the efficacy of various immunotherapeutic strategies, such as viral and cancer vaccines, T cell receptor‐engineered T cell therapies (TCR‐T), and immune checkpoint blockade (ICB).

However, the MHC system also imposes several fundamental challenges for the development of immunotherapies. The extreme polymorphism of classical MHC‐I molecules, though evolutionarily advantageous for population‐level defense against pathogens [2], significantly limits universal treatment design and population‐wide coverage [4]. Additionally, the downregulation or loss of MHC‐I expression, a common immune evasion strategy employed by pathogens and tumors, reduces the efficacy of many immunotherapies by preventing cytotoxic CD8+ T cells from recognizing and eliminating target cells [5]. In allogeneic settings, MHC mismatches between donor immune cells and recipient target cells can trigger adverse immune responses, such as graft rejection or graft‐versus‐host disease (GVHD), complicating the development of off‐the‐shelf cellular therapies [6]. Together, these limitations highlight the urgent need to explore alternative strategies that can overcome these barriers to broaden the reach and robustness of next‐generation immunotherapies.

While classical MHC‐I molecules (HLA‐A, ‐B, and ‐C) have been extensively studied for their roles in antigen presentation and immune regulation, their nonclassical counterparts (HLA‐E, HLA‐F, HLA‐G, CD1, and MR1) remain underappreciated despite their unique roles in immune modulation. Unlike classical MHC‐I, these molecules exhibit limited polymorphism, specialized antigen repertoires, unconventional trafficking behaviors, and the ability to engage both innate and adaptive immunity [7]. Importantly, their expression is often maintained or upregulated in pathological contexts such as infection and cancer [7]. These distinct features position nonclassical MHC‐I molecules as strategically valuable targets to overcome the fundamental limitations of conventional MHC‐restricted therapies, offering new avenues for next‐generation immunotherapies.

This review explores how the special structural and functional features of nonclassical MHC‐I molecules present novel therapeutic opportunities. We first examine the evolutionary adaptations and structural properties that enable the specialized immune functions of these molecules, ranging from unconventional antigen presentation to fine‐tuned immune modulation. We then discuss their distinct receptor interactions and roles in coordinating innate and adaptive immunity within tissues and disease contexts. Finally, we highlight emerging translational strategies targeting nonclassical MHC‐I molecules, while critically evaluating challenges related to delivery, specificity, pharmacodynamics, and safety. By integrating fundamental immunology with therapeutic design, this review aims to provide a strategic framework for harnessing nonclassical MHC‐I molecules to overcome current therapeutic limitations and advance next‐generation immunotherapies.

## 2 Overview of the MHC System

### 2.1 Discovery, Genetic Organization, and Structure of the MHC Molecules

MHC was initially discovered in studies on tumor transplant rejection in a murine model [8]. Follow‐up research highlighted the important role of MHC molecules in orchestrating immune responses [9] and demonstrated that certain MHC alleles are associated with disease susceptibility [10]. The concept of MHC restriction originated from the observation that viral‐specific CTLs eliminated only lymphocytic choriomeningitis virus‐infected target cells sharing the same MHC allotype in mice [11]. Subsequent studies showed that CD8+ and CD4+ T cells are restricted by MHC‐I and MHC‐II molecules, respectively [12, 13]. Later discoveries revealed that MHC molecules bind to foreign peptides and present them for T cell recognition [14, 15, 16]. The crystal structure of HLA‐A2 further revealed a defined peptide binding groove, providing structural insights into this process [17]. Together, these pioneering studies established the MHC as a cornerstone of adaptive immunity.

**FIGURE 1:**
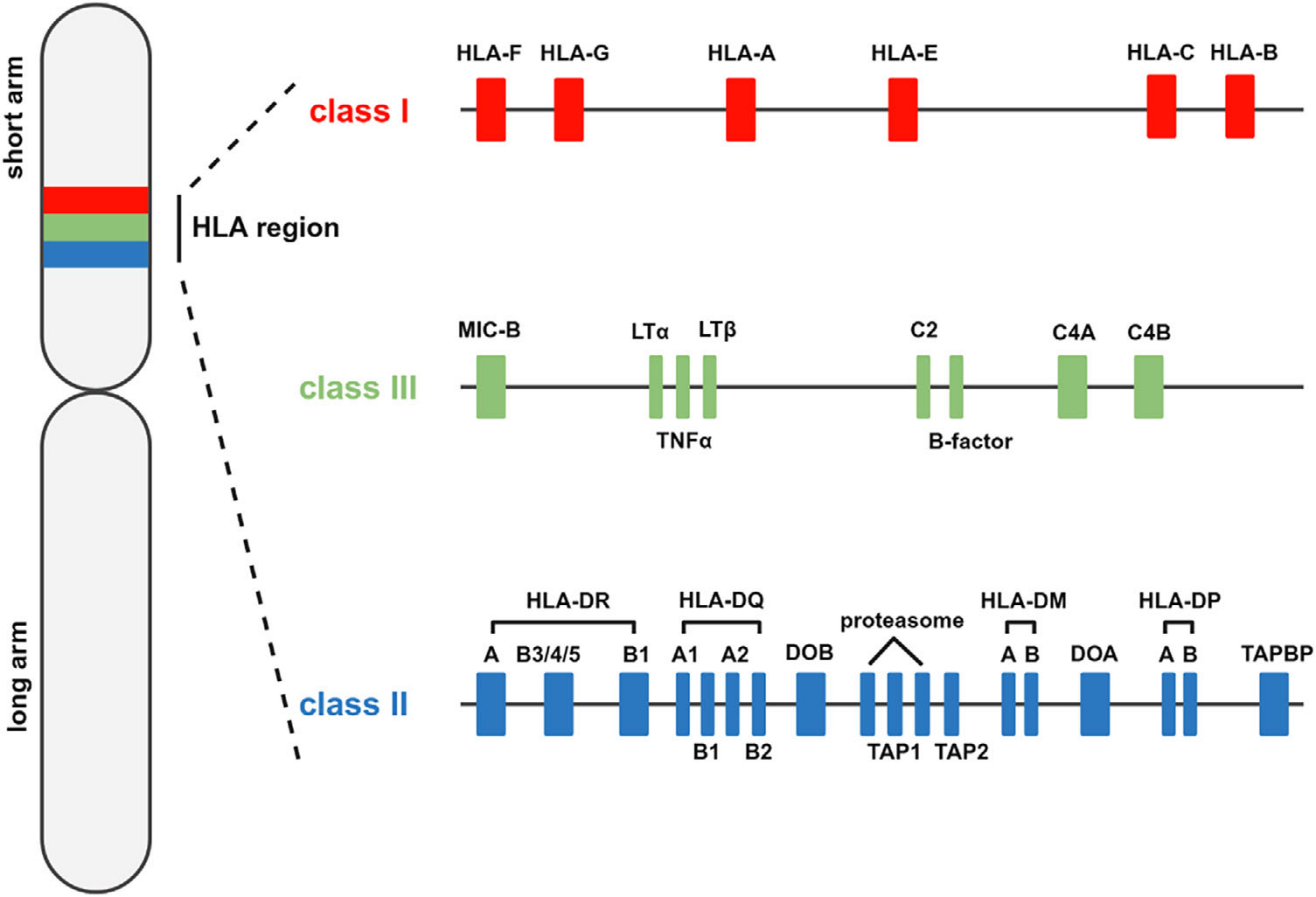
Schematic illustration of human chromosome 6 showing the main MHC genes. This figure illustrates the genomic clustering of major histocompatibility complex (MHC)‐related genes on the short arm of human chromosome 6 (6p21.3). The MHC is divided into three regions—Class I, Class III, and Class II—each region contains genes with distinct immunological roles: the Class I region encodes classical (HLA‐A, ‐B, ‐C) and nonclassical (HLA‐E, ‐F, ‐G) MHC‐I molecules. The Class II region includes HLA‐DP, ‐DQ, and ‐DR molecules, as well as genes encoding proteins involved in antigen processing. The Class III region encodes components of the complement system and inflammatory mediators. Notably, CD1 and MR1 are located outside the MHC locus and are not depicted here. Figure adapted from Ref. [409].

The human MHC gene region, located on the short arm of chromosome 6, contains over 200 genes, many of which are essential to immune system functions. Based on their structure and functions, these genes are categorized into three classes (Figure 1) [18]. Class I genes encode HLA‐I molecules, which are ubiquitously expressed and mainly responsible for presenting endogenous antigens to CTLs [3]. They are further divided into two subsets: the classical HLA‐Ia group (HLA‐A, ‐B, ‐C), characterized by extensive polymorphism, and the nonclassical HLA‐Ib group (HLA‐E, ‐F, ‐G, ‐H), which displays limited polymorphism [19]. The HLA‐II region encodes molecules predominantly expressed on antigen presenting cells (APCs), which are responsible for presenting exogenous antigens to CD4+ T cells, including T helper cells. HLA‐DR, HLA‐DP, and HLA‐DQ represent the major loci and are characterized by allelic diversity [19]. HLA‐DM and HLA‐DO, located in the same region, play essential roles in the peptide loading of HLA‐II proteins [20]. The latter are commonly referred to as nonclassical HLA‐II molecules and exhibit restricted variability. Additionally, the region also contains genes encoding key components involved in HLA‐I peptide loading, including transporter associated with antigen processing (TAP) and tapasin. Class III genes are distinguished from other HLA classes by their role in producing secreted proteins that influence immunity indirectly. These molecules, including complement factors and diverse cytokines, are crucial for immune regulation and defense but do not participate in antigen presentation [21].

Classical MHC molecules exhibit extraordinary genetic polymorphism [22]. This diversity has been selected in evolution by infectious disease over many millennia in humans and over millions of years in other vertebrates [2, 23]. Strong evidence for selection of HLA variants associated with resistance to infection has come from analyses of T cell responses to new pathogens like human immunodeficiency virus (HIV) [24], hepatitis C virus (HCV) [25], and SARS‐CoV‐2 [26], as well as in older ones, including *Mycobacterium tuberculosis* (Mtb) [27]. Conversely, certain HLA alleles have been linked to increased susceptibility to various autoimmune disorders [28]. These findings show a striking contrast to the limited polymorphism in nonclassical HLA‐I and ‐II molecules, implying that they have dominant and highly conserved functions preserved by purifying selection. Yet, given the similarities between these conserved molecules and their polymorphic cousins, there remains an intriguing possibility: if these nonclassical HLA molecules could be modified or primed to present certain foreign immunogens to T cells, alongside their dominant conserved function, they might open up novel and exciting therapeutic options.

Beyond the MHC region, several genes on other chromosomes also encode proteins with structural and functional homology to HLA‐I molecules. Among these are the cluster of differentiation 1 antigen (CD1) and MHC‐related protein 1 (MR1) (also referred to as MHC‐I‐like molecules), which are specialized for the presentation of lipid and metabolite antigens, respectively [29]. Other genes essential for antigen presentation also reside outside the MHC region, including the invariant light chain β‐2‐microglobulin (β2m; chromosome 15) and TAP‐binding protein related (TAPBPR; chromosome 12), a protein vital for HLA‐I peptide loading.

Structurally, the HLA‐I heavy chain is a Type I protein composed of three extracellular domains (α1, α2, and α3), a short transmembrane region, and an intracellular cytoplasmic tail. The α1 and α2 domains form the peptide‐binding groove, whereas the α3 domain associates with β2m [17, 30]. Notably, the peptide‐binding cleft and TCR‐interacting surfaces are the primary sites of the HLA‐I's extensive polymorphism [31, 32]. This genetic diversity has evolved to enable effective immune responses against pathogens, optimizing the immune system's ability to combat infections and threats. The heavy chain typically forms a heterotrimer with β2m and peptide in the ER and then traffics to the cell surface and presents the peptide.

The MHC system is one of the most evolutionarily conserved components of the vertebrate immune system, reflecting its fundamental role in host defense. Homologs of MHC genes have been identified in a wide range of jawed vertebrates, indicating their ancient origin dating back over 400 million years [1, 33]. While the overall genomic organization and specific gene content can vary between species, the core function of MHC molecules—to present peptide antigens to T cells—has been remarkably preserved [1, 2]. Classical MHC‐I molecules across species retain the same structural motifs necessary for peptide binding and interaction with CD8+ T cells, highlighting the evolutionary pressure to maintain effective antigen presentation mechanisms [1]. However, species‐specific adaptations also exist, such as expansion or contraction of certain MHC gene families, differences in allele numbers, and the emergence of unique nonclassical MHC molecules, which reflect the coevolution of the MHC system with diverse pathogen landscapes [2, 33].

### 2.2 The Crucial Role of MHC in the Immune System

**FIGURE 2:**
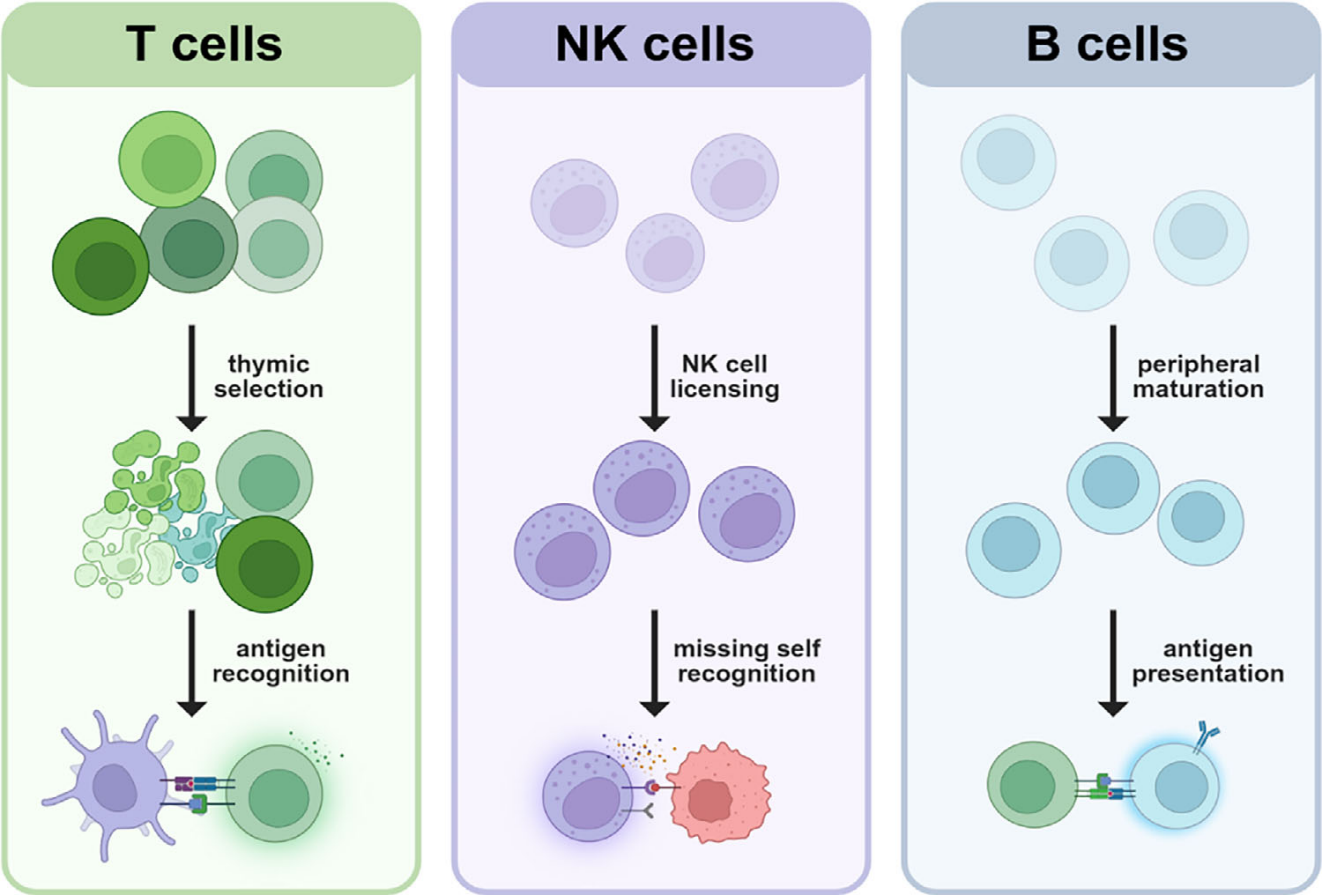
Central roles of MHC molecules in immune cell development and function. The diagram illustrates the pivotal roles of MHC molecules in T cells, NK cells, and B cells. T cells (left panel): MHC molecules are integral to thymic selection and antigen recognition. During thymic selection, T cells undergo positive and negative selection to ensure self‐tolerance and functional competency. MHC molecules present antigens, enabling T cells to recognize and respond to specific pathogens. The interaction between MHC molecules and TCRs is crucial for immune activation. NK cells (middle panel): MHC molecules are involved in NK cell licensing and recognition of “missing self.” Licensing is the process through which NK cells gain functional maturity, often influenced by interactions with MHC Class I molecules on surrounding cells. NK cells recognize cells lacking self‐MHC markers, which may indicate infection or malignancy, prompting the destruction of these compromised cells. B cells (right panel): in B cells, MHC molecules contribute to peripheral maturation and antigen presentation. B cells mature in peripheral lymphoid organs, where MHC molecules aid in the presentation of processed antigens to helper T cells for the activation of humoral immunity. This antigen presentation by B cells is crucial for the production of specific antibodies and the generation of long‐lasting immune memory.

The MHC pathway is indispensable for T cell development in the thymus (Figure 2), where positive and negative selection ensure that emerging T cells are both self‐MHC‐restricted and self‐tolerant [34]. Immature thymocytes expressing TCRs must recognize self‐MHC molecules presented by thymic epithelial cells to receive survival signals [35]. Simultaneously, high‐affinity recognition of self‐peptides leads to deletion of autoreactive clones, a key mechanism to prevent autoimmunity [36]. Notably, TCRs bind to their foreign targets with very low affinity, typically with a Kd of 1–100 µM [37]. This minimizes the chances of functional cross‐reactivity with peptides of similar sequences. Mechanistically, this is facilitated by the immunological synapse—a specialized interface where the TCR engages the peptide–MHC complex surrounded by coreceptors that stabilize the interaction and enhance signal fidelity [38]. Following activation, T cells differentiate into effector and/or memory cells, which then circulate and continuously rely on MHC‐mediated antigen recognition to carry out their functions. Apart from antigen recognition, MHC–TCR interactions also provide essential signals for maintaining T cell survival, memory, and homeostasis [39]. Thus, MHC molecules not only ensure proper T cell development and self‐tolerance but also enable T cells to carry out precise and effective immune surveillance and responses.

In addition, CD1 molecules, which share structural similarity with MHC‐I, present lipid and glycolipid antigens to natural killer T (NKT) cells, enabling rapid responses to microbial and self‐derived lipids. MR1 presents small‐molecule metabolites, including vitamin B derivatives, to mucosal‐associated invariant T (MAIT) cells, which play crucial roles in mucosal immunity and antimicrobial defense. These two nonpolymorphic MHC‐I molecules further extend the range of antigens recognizable by T cells and bridge innate and adaptive immunity.

Alongside the adaptive T cell‐mediated immune responses is the more ancient innate immune system, which responds rapidly and broadly to danger signals, often through pattern recognition receptors. A key player of innate immunity is the natural killer (NK) cell, which is tightly regulated by interactions with MHC/HLA molecules via specific receptors, some of which deliver activating signals while others give inhibitory signals. During development, NK cells undergo a process of “education” or “licensing,” in which engagement of their inhibitory receptors with self‐MHC‐I molecules ensures functional competence and self‐tolerance (Figure 2). A central concept in NK cell biology is the “missing self‐hypothesis,” which proposes that NK cells are activated to kill cells that have lost or downregulated MHC‐I expression—a frequent adaptation of virus‐infected or tumor‐transformed cells, which enables them to evade T cell‐mediated immune attack [40].

NK cell receptors exhibit remarkable specificity for different MHC molecules. Killer cell immunoglobulin‐like receptors (KIRs) allow NK cells to monitor classical HLA‐I molecules with high specificity [41], including HLA‐C and subsets of HLA‐A and ‐B. This recognition relies only minimally on the bound peptide [42]. Different KIRs, containing either two or three domains, deliver either inhibitory or activation signals, with the former usually dominant. This balance regulates NK cell activity in a precise way. The inhibitory receptors leukocyte immunoglobulin‐like receptor subfamily B member 1 (LILRB1) and LILRB2 (also known as immunoglobulin‐like transcript 2 [ILT2] and ILT4) preferentially bind to the nonclassical molecule HLA‐G, contributing to maternal–fetal tolerance and immune modulation [43]. The cluster of differentiation 94 (CD94)/NK Group 2 (NKG2) receptors specifically recognize HLA‐E presenting peptides derived from MHC‐I leader sequences, known as VL9, thereby linking innate recognition to classical MHC expression [44, 45, 46, 47]. Like the KIR receptors, they mediate either inhibitory signaling (via CD94/NKG2A) or activating signaling (via CD94/NKG2C). Overall signaling is biased toward inhibition, as CD94/NKG2A binds to the HLA‐E–VL9 complex with higher affinity. Additionally, the activating NKG2D/DAP10 receptor detects stress‐induced ligands such as MHC‐I polypeptide‐related sequence A (MICA) and MICB [48]. Thus, classical and nonclassical MHC molecules not only shape NK cell development but also orchestrate their functional capability to maintain immune surveillance, protecting the host from infection, malignant transformation, and immune dysfunction.

Although not essential for the early development of B cells or myeloid cells, MHC molecules are indispensable for their functional maturation and immune competence (Figure 2). MHC‐II is critical for the function of mature B cells, enabling them to present antigens to CD4+ T cells, facilitating class‐switch recombination, affinity maturation, and long‐lived antibody responses [49]. Myeloid cells, such as dendritic cells and macrophages, also depend heavily on MHC‐I and MHC‐II to activate naive T cells and shape adaptive immunity. Dendritic cells are specialized in using MHC‐I molecules to cross‐present exogenous antigens to CD8+ T cells, while simultaneously employing MHC‐II molecules to activate CD4+ helper T cells [50]. In contrast, macrophages primarily present antigens via MHC molecules to T cells during chronic inflammation or infection, contributing to sustained immune responses [51]. Thus, while MHC molecules do not determine the lineage identity of these cells, they are fundamental in coordinating immune function and communication between innate and adaptive immunity.

MHC genes are among the most polymorphic in the genome, a feature maintained by balancing selection, particularly through heterozygote advantage and pathogen‐driven pressure [2]. This extreme polymorphism plays a pivotal role in shaping both individual and population‐level immune competence. The genetic diversity of MHC allows different individuals to present a broad array of pathogen‐derived peptides, ensuring that across a population, pathogens are less likely to escape immune detection, thereby providing herd‐level protection against rapidly evolving microbes [52]. However, at the individual level, MHC polymorphism contributes to variations in immune responsiveness, influencing susceptibility to infectious diseases, the risk of autoimmune disorders, and outcomes in organ transplantation [53]. Despite this allelic diversity, the structural framework and functional roles of MHC molecules, including antigen presentation and T cell education, are remarkably conserved across evolution [54]. Together, this unique combination of high polymorphism and deep evolutionary conservation highlights the essential and irreplaceable role of MHC in immune regulation.

### 2.3 MHC‐I in Immunotherapies: Opportunities, Challenges, and Emerging Solutions

**FIGURE 3:**
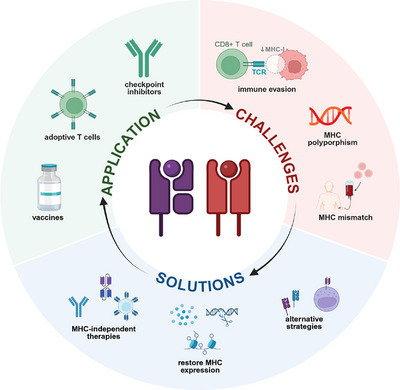
Main applications, challenges, and emerging solutions for MHC‐dependent immunotherapies. MHC‐dependent immunotherapies—such as vaccines, adoptive T cell transfer (e.g., TCR‐T cells), and immune checkpoint inhibitors (e.g., anti‐PD‐1)—have demonstrated significant clinical efficacy. However, their efficacy is often hindered by several MHC‐related challenges, including immune evasion due to MHC‐I downregulation, limited patient coverage stemming from MHC polymorphism, and complications arising from MHC mismatch in allogeneic settings. Emerging strategies to overcome these barriers include MHC‐independent therapies (e.g., CAR‐T, BiTEs), restoration of MHC expression (e.g., IFN‐γ treatment), and alternative approaches (e.g., nonclassical MHC, NK‐based therapies).

The fundamental role of MHC molecules in antigen presentation has made them central to a wide range of immunotherapeutic strategies (Figure 3). In vaccine development, both therapeutic cancer vaccines and prophylactic vaccines against pathogens rely heavily on MHC‐mediated peptide presentation to elicit effective T cell immunity specific for both cell surface and internal antigens. In cancer, personalized vaccines targeting neoantigens have shown promising results in clinical trials [55, 56]. Similarly, for infectious diseases, vaccines designed to target conserved viral or bacterial epitopes are being actively explored against a wide range of pathogens [57, 58]. Adoptive cell therapies (ACTs), such as TCR‐T and other cells expressing TCRs or TCR‐like antibodies, represent another promising class of MHC‐dependent immunotherapies [59]. These approaches involve the transfer of T cells genetically engineered to express tumor‐specific TCRs that recognize antigenic peptides presented by MHC‐I molecules. Several TCR‐T therapies targeting shared tumor antigens, such as NY‐ESO‐1, are currently undergoing clinical trials and have demonstrated encouraging therapeutic potential [60].

In addition, the efficacy of immune checkpoint inhibitors, such as antiprogrammed cell death protein (PD)‐1 and anticytotoxic T‐lymphocyte‐associated protein (CTLA)‐4 therapies, relies on functional MHC‐I presentation to enable the reactivated T cells to recognize and kill tumor cells [61]. Tumors with high neoantigen burden and intact MHC‐I expression tend to respond better to these therapies, while loss of MHC expression is a known resistance mechanism [62]. Beyond oncology and infectious diseases, MHC‐targeted approaches are being developed to regulate immune responses in autoimmunity and transplantation. In autoimmune diseases, the focus is on promoting tolerance and reducing autoimmunity through peptide‐based immunotherapies that engage MHC‐II pathways to induce regulatory T (Treg) cell responses [63]. In transplantation, strategies employed to minimize alloreactivity include MHC matching and the use of MHC‐blocking antibodies or peptide inhibitors to prevent T cell activation against donor antigens [64]. Together, these applications highlight the versatility and central importance of MHC molecules across diverse immunotherapeutic contexts.

However, the biological constraints of MHC present significant challenges to the development and broad application of immunotherapies. A major limitation is the extreme polymorphism of MHC genes, which restricts the application of TCR‐based therapies and peptide vaccines to individuals with matched alleles [4]. This genetic variability complicates the development of universal treatments and limits population‐wide coverage. Additionally, many cancers and chronic infections evade immune recognition by downregulating MHC‐I expression, making these abnormal cells invisible to CD8+ T cells and reducing the efficacy of immunotherapies like ICB and ACT [5]. Beyond immune evasion, the limited peptide‐binding specificity of individual MHC variants restricts the repertoire of presentable antigens. Even highly mutated tumors may display only a few immunogenic epitopes, and computational predictions of MHC binding are often imprecise, making neoantigen vaccine development technically challenging and uncertain [65]. Furthermore, MHC mismatch poses a significant barrier to allogeneic cell therapies such as donor‐derived chimeric antigen receptor (CAR)‐T cells or TCR‐T cells, increasing the risk of rejection or GVHD [6]. While genome editing disrupting MHC expression offers a promising approach to generate universal donor cells, this strategy remains experimental and raises concerns regarding safety and long‐term immune compatibility [66].

To address these challenges, several innovative strategies are under active investigation. One approach aims to bypass MHC dependence entirely—such as using universal CAR‐T cells, TCR mimic antibodies, or bispecific T‐cell engagers (BiTEs) to redirect T cells to tumor targets without requiring MHC‐I‐restricted antigen presentation [66, 67, 68]. In parallel, emerging in vivo engineering approaches are designed to deliver TCRs, CARs, or BiTEs directly as gene therapies or “immune vaccines,” enabling the generation or redirection of effector T cells within the patient [69]. Such in vivo strategies could simplify production, reduce costs, and broaden accessibility. Another strategy focuses on restoring or enhancing MHC‐I function, including upregulating MHC expression with interferon‐γ stimulation or epigenetic modulation, or employing artificial APCs engineered to express a panel of MHC alleles to expand T cells in vitro and then giving them back to patients [70, 71]. Nonclassical MHC molecules offer another promising avenue, as they are less polymorphic, often resistant to downregulation in infection and cancer, and engage both innate and adaptive immune pathways [72]. Additionally, NK cell‐based therapies can take advantage of the loss of MHC‐I expression, a common mechanism of tumor immune evasion, to selectively target cells with reduced MHC‐I levels [73].

In summary, immunotherapies involving MHC are crucial in directing immune responses across a range of clinical applications. While current strategies have achieved notable success, especially in cancer and chronic infection, they are constrained by MHC polymorphism, immune evasion by tumors and pathogens, and risks of adverse immune activation. The integration of MHC‐independent platforms, enhancement of antigen presentation, exploitation of nonclassical MHC molecules, and use of alternative immune recognition systems offers a promising path forward to overcome these limitations and extend the benefits of immunotherapy to a broader patient population.

## 3 HLA‐E

### 3.1 HLA‐E Is Evolutionarily Optimized for VL9 Peptide Presentation

**FIGURE 4:**
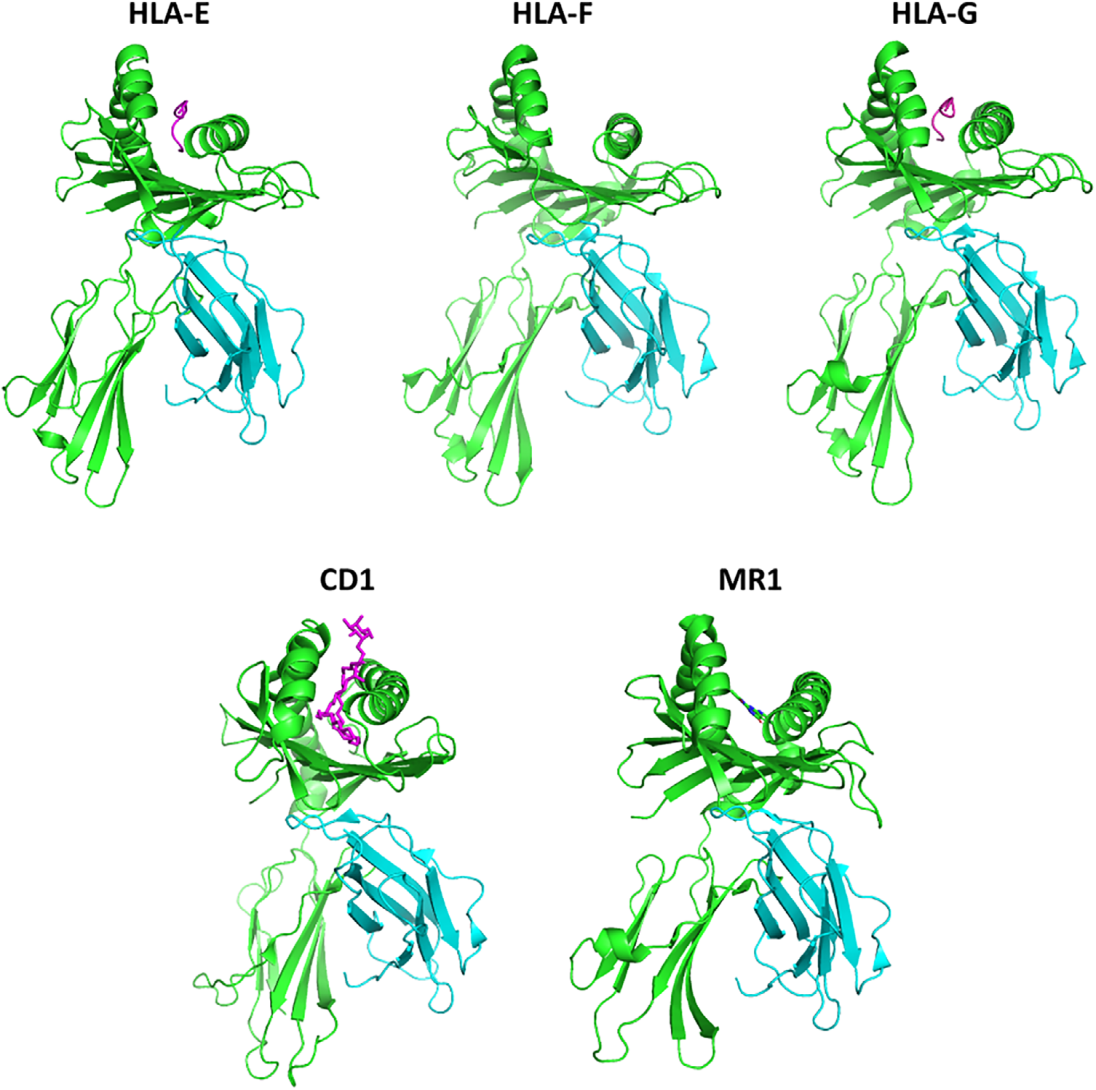
Structure overview of human nonclassical HLA‐I molecules. Representative structures of HLA‐E (PDB ID: 1MHE), HLA‐F (PDB ID: 5KNM), HLA‐G (PDB ID: 1YDP), CD1 (PDB ID: 1ZT4), and MR1 (PDB ID: 4GUP). The HLA heavy chains (green) are shown in complex with β2‐microglobulin (cyan). The distinct ligand types are highlighted: peptide for HLA‐E (VMAPRTVLL) and HLA‐G (RIIPRHLQL), lipid for CD1d (α‐GalCer), and metabolite for MR1 (5‐OP‐RU). HLA‐F is depicted in a peptide‐free form. The images were created from the PDB data cited above using the PyMOL Molecular Graphics System, Version 3.1.6.1, Schrödinger, LLC.

One of the key distinctions between HLA‐E and classical MHC‐Ia molecules lies in their peptide repertoire and polymorphism (Figure 4). Classical MHC‐Ia molecules are highly polymorphic and can present a diverse repertoire of peptides to CD8+ T cells, facilitating immune recognition of diverse antigens. In contrast, the nonclassical HLA‐Ib molecule HLA‐E is nonpolymorphic, with only two predominant alleles (HLA‐E*01:01 and HLA‐E*01:03) [74]. They share an identical peptide‐binding groove and differ by a single residue at position 107 [75]. HLA‐E presents a narrow range of peptides to NK cells, mostly derived from the signal sequences of classical MHC‐I molecules, typically VMAPRTL(L/V/I)L (VL9) [75, 76, 77]. The HLA‐E binding groove is tailored for VL9, with key contributions from amino acid residues at positions 67, 143, 147, 152, and 156 [75].

This limited polymorphism and VL9‐dominated peptidome of HLA‐E reflect its primary role in presenting HLA‐Ia leader sequence‐derived VL9 peptides for recognition of NK cells and a subset of activated CD8+ T cells [44, 78, 79]. The HLA‐E–VL9 complex is a ligand for CD94–NKG2A and CD94–NKG2C receptors on different subsets of NK cells [80]. The former binds with higher affinity and suppresses NK cell activation [81], while the latter transmits an activating signal to NK cells [82]. Together, these opposing interactions fine‐tune NK cell responsiveness, allowing HLA‐E to serve as a “missing‐self” sensor that protects healthy cells from NK cell‐mediated lysis but not abnormal cells without intact MHC‐I antigen presentation pathways [44, 46]. Furthermore, engagement of HLA‐E with the CD94/NKG2A receptor on a small population of activated CD8+ T cells delivers inhibitory signals that suppress T cell activation and cytotoxicity, a mechanism recognized as essential for maintaining immune tolerance [78, 79] (Table 1).

**TABLE 1:** Receptors for nonclassical MHC‐I molecules and corresponding immune cell subsets.

MHC‐I	Immune cells	Receptors	Inhibitory	Activating	References
HLA‐E	NK	NKG2A/CD94	X		[83]
		NKG2C/CD94		X	[84, 85]
	NKT cells	NKG2A/CD94	X		[83]
	CD8+ T cells	NKG2A/CD94	X		[78, 79, 83, 86]
		NKG2C/CD94		X	[85]
		TCR		X	[87]
	CD4+ T cells	NKG2A/CD94	X		[86, 88]
		NKG2C/CD94		X	[89]
		TCR		X	[90]
	γδ T cells	NKG2A/CD94	X		[91]
		NKG2C/CD94		X	[92]
HLA‐G	NK cells	LILRB1	X		[93]
		KIR2DL4	X	X	[94, 95]
		CD8	X		[43]
	NKT cells	LILRB1	X		[96]
	CD8+ T cells	LILRB1	X		[93]
		CD8	X		[97]
		KIR2DL4	X		[93]
	CD4+ T cells	LILRB1	X		[93]
	γδ T cells	LILRB1	X		[98]
	Dendritic cells	LILRB1	X		[93]
		LILRB2	X		[99]
	B cells	LILRB1	X		[93]
	Monocytes/macrophages	LILRB1	X		[93]
		LILRB2	X		[93]
	Neutrophil	LILRB2	X		[100]
	Myeloid‐derived suppressive cells	LILRB1	X		[93]
		LILRB2	X		[101]
HLA‐F^a^	NK cells	KIR3DS1		X	[102]
		KIR3DL2	X		[103]
	CD8+ T cells	KIR3DL2	X		[103]
	CD4+ T cells	KIR3DL2	X		[103]
CD1^b^	iNKT cells	Vα24‐Jα18/Vβ11^c^		X	[104]
	Type II NKT	αβTCR		X	[104]
	Conventional T cells	αβTCR		X	[105]
	γδ T cells	γδTCR		X	[106, 107, 108]
MR1	MAIT	Semi‐invariant αβTCR		X	[109]
	Conventional T cells	αβTCR		X	[110]
	γδ T cells	γδTCR		X	[111]

Abbreviations: CD1, cluster of differentiation 1; KIR, killer cell immunoglobulin‐like receptor; LILRB1/2, immunoglobulin‐like receptor subfamily B member 1/2 (also known as Immunoglobulin‐like transcript 2 [ILT2] and ILT4); MAIT, mucosal‐associated invariant T cell; MR1, the MHC‐I‐related molecule 1; NK, natural killer cell; NKT, natural killer T cell; TCR, T‐cell receptor; X indicates a reported interaction. Receptor–ligand pairs are shown using standard abbreviations: HLA, human leukocyte antigen; αβTCR, alpha‐beta TCR; γδTCR, gamma‐delta TCR.

^a^ HLA‐F has been reported to interact with additional receptors, but their functional roles on specific immune cell subsets remain unclear and are therefore not included here. Notably, KIR3DS1 can bind both peptide‐loaded HLA‐F and open conformers, whereas other receptors listed here have only been shown to recognize the open conformer.

^b^ NKT cells exclusively recognize CD1d, and conventional T cells exclusively recognize CD1a, CD1b, or CD1c. In contrast, γδ T cells have been reported to recognize CD1a through CD1d.

^c^ The canonical TCR α‐ and β‐chain rearrangements characteristic of human invariant NKT (iNKT) cells.

The structural features of HLA‐E and its unconventional trafficking upon surface arrival further support its major role in regulating NK cells. Surface expression of HLA‐E requires the VL9 peptide [76, 112], which promotes the proper refolding of HLA‐E into a compact and stable form. This enables HLA‐E to pass the proofreading checkpoints mediated by tapasin and TAPBPR, facilitating its exit from the ER [113]. As VL9 is derived from the signal sequences of classical MHC‐Ia molecules, the surface level of HLA‐E serves as an effective indicator of HLA‐Ia expression. Despite VL9 being ubiquitously expressed, HLA‐E is not saturated with endogenous VL9 under normal conditions [114]. Besides, the peptide‐receptive HLA‐E/β2m dimers are more stable than their HLA‐Ia counterparts [115]. Such relative abundance and relatively rigid structure of intracellular peptide‐receptive HLA‐E likely enhances its sensitivity to fluctuations in HLA‐Ia expression, thereby ensuring its capability to modulate NK cell responses [116].

Unlike classical HLA‐Ia molecules, which are mostly stable on the cell surface, HLA‐E is rapidly internalized following its arrival at the plasma membrane, with a surface half‐life of around 30 min [114]. This instability arises in part from the lower binding affinity of VL9 than most HLA‐Ia peptides, which results in a less stable HLA‐E–VL9 complex, even though VL9 is considered the strongest HLA‐E‐binding peptide [116, 117, 118, 119]. The fast internalization of HLA‐E is further facilitated by its cytoplasmic tail [114], with a unique motif driving rapid clathrin‐mediated endocytosis, in contrast to the pathways used by classical HLA‐I molecules [120]. Together, these unique features allow HLA‐E to reflect the cellular status of HLA‐Ia molecules in a dynamic manner, enabling the timely detection of abnormal cells by NK cells.

Under abnormal conditions such as pathogen infection, the suppression of HLA‐Ia surface expression and disruption of the classical antigen presentation machinery are common strategies employed to evade T cell recognition [5]. In contrast, HLA‐E level is often maintained or even upregulated [121, 122], helping target cells avoid NK cell‐mediated killing and suppress T cell responses. HLA‐E trafficking is often regulated differently from that of classical HLA‐I molecules in these cases, as shown for Mtb [123], human cytomegalovirus (HCMV) [121, 124], and HIV [122, 125, 126]. To facilitate immune escape, tumor cells not only upregulate their own HLA‐E surface level for self‐protection [83, 127, 128], but also secrete soluble HLA‐E, thereby extending the protection to neighboring cells [129, 130, 131].

Despite sequence variations, MHC‐E homologs across species exhibit striking functional and structural conservation [115, 132], underscoring the critical role of the ancient “missing‐self” recognition system in immune regulation. Similar to HLA‐E, the murine homolog quality assurance 1 (Qa‐1) is nonpolymorphic, with only four known alleles identified to date [132]. Qa‐1 shares a similar peptide‐binding specificity with HLA‐E [75, 112, 133, 134, 135], preferentially presenting the Qdm peptide (AMAPRTLLL) derived from the signal sequences of H‐2D/L (murine MHC‐I) [136, 137], a functional parallel to the HLA‐E/VL9 interaction for NK cell regulation. Similar to VL9, Qdm binds to Qa‐1 with moderate affinity, leading to relatively low surface stability of the Qa‐1/Qdm complex [138]. Such reduced stability may stem from the replacement of tryptophan with serine at positions 143 and 147 [139], as is observed in HLA‐E [75]. Mamu‐E (Rhesus monkey MHC‐E) has approximately 30 alleles, which are slightly more diverse than HLA‐E but still markedly less polymorphic than MHC‐Ia molecules [140]. Besides, while HLA‐E is essentially dimorphic, Mamu‐E gene duplications are very common with more polymorphism [141], although the peptide binding groove of Mamu‐E remains well conserved [140]. Mamu‐E resembles HLA‐E in both sequence and structure, especially in the peptide‐binding groove region [115]. Therefore, investigation of HLA‐E orthologues extends the potential peptide repertoire of HLA‐E, reveals its conserved structural and functional features, and offers relevant nonhuman primate (NHP) models.

### 3.2 Unique Features of HLA‐E Also Facilitate Unconventional Peptide Presentation

Beyond its well‐characterized role in presenting leader peptides from classical HLA‐Ia molecules, HLA‐E can also present pathogen‐ and tumor‐derived peptides and activate CD8+ T cells [87]. Emerging evidence indicates that these unconventional HLA‐E‐restricted CD8+ T cell responses arise broadly in bacterial, viral, and tumor contexts, though this area remains understudied. In Mtb infection, HLA‐E can present Mtb‐derived peptides for the recognition of CD8+ T cells, controlling bacterial growth in infected individuals [142, 143]. Among the Mtb‐derived peptides that can bind to HLA‐E and activate CD8+ T cells, Mtb44 (RLPAKAPLL) is the most striking [144], with peptide binding affinity comparable to VL9 [115]; however, it is not actually very immunogenic [145]. The IL9 peptide (IMYNYPAML) binds well to HLA‐E and is immunodominant [117, 146]. Another Mtb‐derived peptide, Rv0634A19‐29 (EIEVDDDLIQK), is broadly recognized by HLA‐E‐restricted CD8+ T cells across donors [146]. Despite having a highly charged rather than hydrophobic sequence, it is immunogenic, suggesting it may bind HLA‐E in an unconventional manner [146]. Similarly, in *Salmonella typhi* (*S.typhi*) infection, HLA‐E presents bacterial peptides that activate CD8+ T cells, leading to sustained inhibition of bacterial pathogenesis [147, 148]. In viral infections, HLA‐E‐restricted responses have been documented against Epstein–Barr virus (e.g., the BZLF139‐47 peptide) [149], HCV (the core‐derived YLLPRRGPRL peptide) [122, 150], SARS‐COV2 [151], and HCMV (via UL40‐encoded VL9‐mimics) [149, 152]. H‐2 Qa‐1‐restricted presentation of pathogen‐derived peptides has also been reported in *Mtb* [153], *Listeria monocytogenes* [154], *S.typhi* [155], and influenza infections in mice [156].

Mamu‐E can present peptides derived from simian immunodeficiency virus (SIV) and elicit protective CD8+ T cell responses in Rhesus macaques (RMs) vaccinated with a Rhesus cytomegalovirus (RhCMV) 68‐1‐vectored vaccine containing recombinant SIV genes [157]. Similarly, RhCMV68‐1‐vectored vaccines expressing antigens derived from Mtb, malaria, or hepatitis B virus (HBV) can elicit MHC‐E‐restricted CD8+ T cell responses that inhibit pathogenesis in RMs [158, 159, 160]. HLA‐E‐restricted CD8+T cell responses targeting the HIV Gag‐derived RL9 peptide (RMYSPTSIL) and the Rev peptide IL9 (ILVESPTVL) can be efficiently induced in vitro, leading to suppression of HIV replication [161, 162]. Besides, HLA‐E‐restricted T cell responses specific for the Gag peptide KAFSPEVIPML, which is also presented by HLA‐B*57, have been described [163]. Some other peptides derived from HIV have also been shown to bind HLA‐E and enhance its surface stability [164]. Given the strong similarities between Mamu‐E and HLA‐E in expression, sequence, and peptide binding properties [115, 140, 157], effective HLA‐E‐restricted CD8+ T cell responses may likewise play a role in HIV control in vivo, though further studies are needed to validate this.

Beyond generating unconventional effector CD8+ T cell responses, HLA‐E also serves as a restriction element for a specialized lineage of CD8+ Treg cells that maintain immune homeostasis and self‐tolerance [165]. In mice, Qa‐1‐restricted CD8+ Treg cells recognize self‐peptides presented by activated CD4+ T cells [166, 167], enabling them to suppress T follicular helper cells and autoreactive CD4+ T cell subsets, thereby maintaining self‐tolerance [168, 169]. Disruption of Qa‐1 impairs CD8+ Treg function and promotes autoimmunity [170]. Although their human counterparts remain less well defined, emerging evidence suggests that HLA‐E‐restricted CD8+ Treg cells contribute to immune tolerance [171]. Together, these findings indicate that HLA‐E shapes immune homeostasis by regulating both effector and regulatory CD8+ T cell subsets, thereby enabling precise, context‐dependent control of adaptive immune responses.

Release of the ER reservoir of HLA‐E, dependent on the supply of VL9 peptide, rapidly increases its surface expression. Then, the rapid surface turnover of HLA‐E results in its accumulation within endosomal compartments, particularly late endosomes and recycling endosomes [114]. HLA‐E/β2m dimers demonstrate higher stability and a higher tolerance for acidic environments than HLA‐Ia molecules [115, 172]. These properties likely facilitate peptide exchange within endosomes and promote efficient recycling of HLA‐E back to the cell surface [120]. Besides, the relatively broad and rigid peptide‐binding groove of HLA‐E allows it to accommodate and present a diverse array of pathogen‐derived peptides from endosomal compartments [115]. The relatively low peptide‐binding affinity, limited surface expression, and fast surface turnover of HLA‐E together help to prevent excessive T cell exhaustion, contributing to better tumor control [173]. Thus, HLA‐E's unique adaptation for presenting VL9 peptides to CD94/NKG2 receptors on NK and T cells also enables its nonclassical function of presenting pathogen‐derived peptides through an unconventional endosomal pathway.

### 3.3 HLA‐E Is a Promising Target for Next‐Generation Immunotherapies

Pathogen‐derived epitopes presented by HLA‐E offer several advantages that render them attractive targets for vaccine‐elicited CD8+ T cell responses. The lack of genetic polymorphism is the biggest advantage, as it would enable universal vaccines or T cell‐based therapies. Also, many pathogens downregulate the surface expression of HLA‐Ia but not HLA‐E to preserve the HLA‐E/NKG2A axis [174]. Compared with classical MHC‐Ia molecules, HLA‐E presents lower‐affinity peptides, which is not necessarily a disadvantage. This characteristic leads to the rapid surface turnover of HLA‐E, making it less likely to induce T cell exhaustion than the persistent, high‐stability interactions of HLA‐Ia/peptide/TCR complexes [173]. Moreover, HLA‐E could modulate both NK cells and T cells simultaneously, offering potential for dual regulation of the innate and adaptive immune responses. The recent success of the RhCMV68‐1‐vectored vaccine in eliciting protective MHC‐E‐restricted CD8+ T cell responses against multiple infections in RMs strongly suggests that exploiting HLA‐E could be a viable strategy for developing vaccines that elicit protective CD8+ T cells against a broad spectrum of infections [157, 159, 160].

Despite these compelling advantages, translating HLA‐E‐restricted antigen presentation into precise and safe vaccine strategies presents substantial biological and technical challenges. HLA‐E is structurally optimized to present the highly conserved VL9 peptide, which dominates its peptide repertoire [75, 76, 77], creating an intrinsic challenge for the efficient loading of pathogen‐ or tumor‐derived peptides that generally bind with lower affinity. CMV‐based vectors could alter antigen processing and trafficking pathways to enable unconventional peptide loading onto HLA‐E [121, 124, 175]. However, it may also promote presentation of atypical or self‐peptides. What controls which peptides are displayed by HLA‐E remains poorly understood and the relatively rare naturally occurring HLA‐E‐restricted T cells are not well characterized. Consequently, vaccine‐elicited HLA‐E‐restricted T cell responses could be cross‐reactive, highlighting the need for careful evaluation of potential off‐target effects. Improving delivery and specificity will therefore require rational peptide design, controlled antigen expression, vector refinement, and more precise delivery strategies [176]. In addition, CMV‐based vaccination induces HLA‐E upregulation, which may be necessary for robust HLA‐E‐restricted T cell priming [175], but could simultaneously enhance NK inhibition. Combining vaccination with NKG2A blockade may help decouple T cell activation from NK suppression and improve therapeutic efficacy.

Immune checkpoint pathways play crucial roles in immune tolerance but are often hijacked by tumors to evade immune surveillance [177]. While current ICB therapies have revolutionized cancer treatment, their effects remain largely limited to CD8+ T cells, potentially leading to low response rates—particularly in patients with a low tumor mutation burden [177]. ICB treatment of tumors can result in downregulation of HLA‐A, ‐B, and ‐C, impairing T cell recognition, while simultaneously upregulating HLA‐E to engage the inhibitory receptor NKG2A, suppressing both NK and T cell activity [178]. Targeting this coinhibitory HLA‐E/NKG2A axis presents a unique therapeutic opportunity to unleash the cytotoxic functions of both NK cells and T cells, allowing for simultaneous restoration of innate and adaptive immunity. Emerging clinical data demonstrate promising results with NKG2A blockade [179, 180] (Table 2), particularly in combination with existing immunotherapies like PD‐L1 blockade [181]. Therefore, this dual mechanism of action, bridging both arms of the immune system, positions the HLA‐E/NKG2A pathway as a compelling target for next‐generation immunotherapies against cancer and persistent viral infections.

**TABLE 2:** Clinical trials for immunotherapies targeting nonclassical MHC‐I molecules.

Target	Drug/intervention	Mechanism	Clinical phase	Condition	Clinical trials
HLA‐E	NKG2A receptor blockade				
	Monalizumab/HY‐0102	Anti‐NKG2A	Phase I/II	Multiple cancers	NCT06094777, NCT06892223, EUCTR2020‐005902‐24‐IT
	Monalizumab + MEDI5752	Anti‐NKG2A + anti‐PD‐1/CTLA‐4	Phase II	Metastatic MSI/dMMR cancers	NCT06152523
	Monalizumab + durvalumab	Anti‐NKG2A + anti‐PD‐1	Phase I/II	Multiple cancers	NCT06503614, NCT02671435, NCT03833440, NCT06769126, NCT07146230, NCT05221840, CTR20221537
	Monalizumab + durvalumab + chemotherapy	Anti‐NKG2A + anti‐PD‐1 + chemotherapy	Phase II	(Non‐)small cell lung cancer	^b^NCT05903092, NCT05061550
	S095029 + Sym021/pembrolizumab/cemiplimab	Anti‐NKG2A + anti‐PD‐1	Phase I/II	Multiple cancers	NCT05162755, NCT06116136, NCT06162572
	Monalizumab + cetuximab	Anti‐NKG2A + anti‐EGFR	Phase III	Head and neck cancer	^b^NCT04590963
	Monalizumab + trastuzumab	Anti‐NKG2A + anti‐HER2	Phase II/III	Metastatic HER2+ breast cancer	NCT04307329
	Monalizumab + cetuximab+PD(L)1 inhibitor	Anti‐NKG2A + anti‐EGFR + anti‐PD(L)1	Phase I/II	Head and neck cancer	NCT02643550
	HLA‐E‐restricted T cell vaccines				
	VIR‐1388	T‐cell‐based vaccine	Phase I	HIV	NCT05854381
	VIR‐1111	T‐cell‐based vaccine	Phase Ia	HIV	NCT04725877
	RhCMV‐based T cell vaccine	HLA‐E‐restricted CD8+ T cell vaccines	Rhesus macaques	HIV, Mtb, malaria, HBV, cancers	Preclinical
HLA‐F	Anti‐HLA‐F antibody	Anti‐HLA‐F	Rag2−/− mice	Cancer model	Preclinical
HLA‐G	Direct HLA‐G targeting				
	UCB4594/RO7515629	Anti‐HLA‐G	Phase I/II	HLA‐G+solid tumors	NCT06380816, ^b^NCT05769959
	TTX‐080(+pembrolizumab/cetuximab + FOLFIRI)	Anti‐HLA‐G(+Anti‐PD‐1/EGFR + chemotherapy)	Phase I	Advanced cancers	NCT04485013
	IVS‐3001	HLA‐G‐CAR‐T cells	Phase I/II	Advanced HLA‐G+solid tumors	NCT05672459
	CAR001	HLA‐G‐CAR.BiTE allogeneic γδ T cells	Phase I/II	Solid tumors	NCT06150885
	JNJ‐78306358	HLA‐GxCD3 T cell engager (BiTE)	Phase I	Advanced solid tumors	NCT04991740
	SOB100	HLA‐G‐targeted exosome therapy	Phase I	N/A	NCT07219940
	Indirect HLA‐G targeting: LILRB2(ILT4)/LILRB1(ILT2) receptor blockade				
	MK‐4830 + pembrolizumab/quavonlimab	Anti‐LILRB2 + anti‐PD‐1/CTLA‐4	Phase I/II	Multiple tumors	NCT04165070, NCT05319730, NCT04626518, NCT04938817, NCT04895722
	IO‐108 + pembrolizumab/tislelizumab	Anti‐LILRB2 + anti‐PD‐1	Phase I	Advanced solid tumors	NCT05508100, NCT05054348
	OR502/ES009	Anti‐LILRB2	Phase I/II	Advanced solid tumors	NCT06090266, NCT06007482
	CHS‐1000(+toripalimab‐tpzi)	Anti‐LILRB2(+anti‐PD‐1)	Phase I	Solid tumors	NCT06389526
	JTX‐8064(+pimivalimab)	Anti‐LILRB2(+anti‐PD‐1)	Phase I/II	Advanced refractory solid tumors	NCT04669899
	CDX‐585	Anti‐LILRB2/PD‐1	Phase I	Advanced malignancies	NCT05788484
	SPX‐303	Anti‐LILRB2/PD‐L1	Phase I	Solid tumors	NCT06259552
	AGEN1571(+balstilimab/botensilimab)	Anti‐LILRB1(+anti‐PD‐1/CTLA‐4)	Phase I	Advanced solid tumors	NCT05377528
	SAR444881(+pembrolizumab/cetuximab)	Anti‐LILRB1(+anti‐PD‐1/EGFR)	Phase I/II	Advanced solid tumors	^b^NCT04717375
	NGM707(+pembrolizumab)	Anti‐LILRB1/LILRB2(+anti‐PD‐1)	Phase I/II	Solid tumors	NCT04913337
	IOS‐1002(+pembrolizumab)	Anti‐LILRB1/LILRB2/KIR3DL1(+anti‐PD‐1)	Phase I	Advanced solid tumors	NCT05763004
	PF‐07826390(+pembrolizumab)	Anti‐LILRB1/LILRB2(+anti‐PD‐1)	Phase I	Advanced solid tumors	^b^NCT06546553
CD1	CD1 ligands and agonists				
	KRN7000(α‐GalCer)	iNKT cell agonist	Phase I/II	Chronic HBV/HCV infection	NCT00363155, NCT00352235
	PRECIOUS‐01(IMM60 + NY‐ESO‐1)	Synthetic iNKT agonist + peptide vaccine	Phase I	NY‐ESO‐1+ cancers	NCT04751786
	DC‐KRN7000 + lenalidomide	α‐GalCer‐loaded DC + lenalidomide	Phase I/II	Multiple myeloma	NCT00698776
	BDCA‐1 + myDC	Autologous CD1c + myeloid DC injection	Phase I	Advanced melanoma	NCT03747744
	CAR‐NKT cell therapies				
	GINAKIT2 (GD2‐CAR NKT)	Autologous GD2‐CAR NKT cells	Phase I	Children with neuroblastoma	NCT03294954
	GKL‐006 + TACE	Autologous CAR‐NKT cell therapy + chemotherapy	Phase II	Unresectable hepatocellular carcinoma	NCT07209813
	ANCHOR (KUR‐502)	Allogeneic CD19‐targeted CAR‐NKT cells	Phase I	B cell lymphomas	NCT05487651
	GT719	Allogeneic CD19‐targeted CAR‐NKT cells	Phase I	B cell malignancies	NCT07131254
	hCD19.IL15.CAR‐iNKT	Allogeneic CD19‐targeted CAR‐iNKT cells with IL‐15	Phase I	B cell malignancies	NCT04814004
	CGC729	Allogeneic CD70‐targeted CAR‐NKT cells	Phase I	Advanced RCC and solid tumors	NCT06182735, NCT06394622
	CGC738	Allogeneic CD70‐targeted CAR‐NKT cells	Phase I	Advanced RCC and solid tumors	NCT06728189, NCT06870279
	Anti‐CD1a CAR‐T	CAR‐T cell infusion	Phase II	CD1a+ acute leukemia/lymphoma	NCT05745181
	NKT cell Infusions				
	agenT‐797 + balstilimab + botensilimab + ramucirumab + paclitaxel	iNKT cell therapy + anti‐PD‐1 + anti‐CTLA‐4 + anti‐VEGFR2 + chemotherapy	Phase II	Advanced esophageal, gastric, or gastro‐esophageal junction adenocarcinoma	NCT06251973
	iNKT cell infusion	Allogeneic iNKT cell infusion	N/A	Advanced pancreatic cancer	NCT07055568
	iNKT cells + PD‐1 + CD8 + T cells	Autologous iNKT cells and PD‐1 + CD8 + T cells infusion	Phase I/II	Advanced solid tumors	NCT03093688
	iNKT cells + anti‐PD‐1 antibody(+regorafenib)	Autologous iNKT cell infusion+anti‐PD‐1(+anti‐angiogenesis)	Phase II	Progressed hepatocellular carcinoma	NCT05962450
	NKT cell infusion	Autologous NKT cell infusion	Phase I/II	Multiple cancers	NCT02562963, NCT01801852
	NK + NKT cells infusion	Autologous NK + NKT cell infusion	Phase I/II	Non‐small cell lung cancer	NCT03198923
MR1	MR1‐blocking antibodies	Anti‐MR1	Murine model	MCA‐induced sarcoma	Preclinical
	5‐OP‐RU + IAV vaccine	MR1 ligand 5‐OP‐RU + viral vaccines	Murine model	Multiple infections	Preclinical

Abbreviations: CAR, chimeric antigen receptor; CD1, cluster of differentiation 1; CTLA‐4, cytotoxic T lymphocyte‐associated protein 4; dMMR, deficient mismatch repair; EGFR, epidermal growth factor receptor; HER2, human epidermal growth factor receptor 2; IAV, influenza A virus; KIR, killer cell immunoglobulin‐like receptor; LILRB1/2, immunoglobulin‐like receptor subfamily B member 1/2 (also known as immunoglobulin‐like transcript 2 (ILT2) and ILT4); MCA, 3‐methylcholanthrene; MR1, the MHC‐I‐related molecule 1; MSI, microsatellite instability; NK, natural killer cell; NKG2A, natural killer Group 2 member A; NKT, natural killer T cell; PD‐1, programmed cell death protein 1; RCC, renal cell carcinoma.

^a^ *Data source*: https://clinicaltrials.gov/.

^b^ These clinical trials are suspended or terminated.

As HLA‐E surface level depends on the availability of the VL9 peptide, some viruses have evolved strategies to preserve or even enhance the VL9 peptide repertoire to maintain high HLA‐E levels. The best‐studied example is HCMV, which encodes its own VL9 peptide within the signal sequence of the UL40 protein [121]. This generates VL9 in a TAP‐independent manner, thereby evading the HCMV genes that block classical antigen and VL9 processing. Therefore, disrupting VL9 peptide processing in cancer cells offers a potential strategy to downregulate HLA‐E on abnormal cells, unleashing robust immune responses. Cancer therapies targeting endoplasmic reticulum aminopeptidases (ERAPs), which are critical for the final stage of VL9 peptide trimming, have shown effective antitumor effects [182]. As ERAP proteins also shape the classical MHC‐I immunopeptidome [183], they serve as promising targets for modulating both innate and adaptive immunity. Another approach explores antibodies specific for the HLA‐E–VL9 complex to block its interaction with CD94/NKG2A, which have been shown in vitro in humans and in a humanized mouse model to enhance the elimination of infected cells by activating both NK cells and CD8+ T cells [162]. However, this strategy may also diminish the beneficial effects of NKG2C+ NK cells, particularly in CMV+ individuals in whom NKG2C+ NK cells are often expanded [184, 185].

While these studies highlight several distinct strategies to therapeutically target the HLA‐E pathway, each has some inherent limitations and challenges. Blocking antibodies against CD94/NKG2A or the HLA‐E–VL9 complex can rapidly release inhibitory signaling but may require continuous administration. HLA‐E engages with multiple receptors, so careful combination strategies should be considered. Disruption of HLA‐E functions might also inhibit the regulatory functions of CD8+ Treg cells, leading to unwanted activation of T cells and B cells. The broad expression of HLA‐E raises concerns that systematic blockade could disrupt NK/T cell tolerance and increase the risk of unwanted damage to healthy tissues. Similarly, cell‐based approaches, such as CAR‐T/NK cells and vaccines, also carry risks of off‐tumor effects due to the universal expression of HLA‐E. Direct manipulation of HLA‐E cargo offers broader immune reprogramming opportunities, but might also reshape classical MHC‐I immunopeptidomes, as they share antigen processing machinery. Together, these considerations underscore that successful therapeutic exploitation of HLA‐E will likely depend on carefully balanced, context‐dependent combination strategies that maximize immune activation while preserving immune tolerance.

## 4 HLA‐F

### 4.1 HLA‐F Is Evolutionarily Optimized for Its Special Role in Immune Regulation

HLA‐F is a nonclassical MHC‐I molecule whose enigmatic functions are only beginning to be understood (Figure 4). HLA‐F plays specialized roles in immune regulation, interacting with multiple NK cell receptors on NK cells and a subset of T cells [186] (Table 1). Emerging evidence highlights its protective functions in pregnancy and the peripheral nervous system [102, 187, 188], as well as its potential to inhibit viral replication [189]. Notably, HLA‐F may associate with open‐conformed HLA‐I molecules and could potentially facilitate their cross‐presentation [190, 191], suggesting a unique role in immune surveillance. Despite these advances, HLA‐F's full biological significance and therapeutic potential await further exploration.

Unlike classical MHC‐I molecules, the surface expression of HLA‐F is highly selective, primarily observed at the maternal–fetal interface and on immune cells upon activation [192, 193, 194, 195]. This conditional expression acts as a dynamic immunological checkpoint, facilitating context‐specific immune regulation. HLA‐F is constitutively expressed across various tissues and cell types, but is predominantly retained intracellularly [196], enabling rapid mobilization for timely immune modulation while minimizing unintended immune activation [195]. ER export of HLA‐F is not affected by peptide binding [192], ensuring surface mobilization occurs only in response to immune activation or cellular stress. Notably, HLA‐F is quickly translocated to the surface in activated lymphocytes but not in Treg cells [193], highlighting its role in balancing immune tolerance and controlled responses, though the precise regulatory mechanisms remain unclear. Though the surface half‐life of HLA‐F remains uncharacterized, its structural stability in a peptide‐free conformation and its role in sustained immune inhibition likely confer better surface stability compared with classical MHC‐I molecules. Together, these unique transport features and precisely regulated expression patterns position HLA‐F as a dynamic yet tightly controlled immune modulator.

HLA‐F does not typically associate with peptides in the same manner as classical MHC‐I molecules, but is mostly found to exist in an open conformer state [192, 196]. Such open conformation may facilitate its association with HLA‐I heavy chains on the cell surface [190], which serve as ligands for a range of activating and inhibitory immune receptors [186], thus delivering modulatory signals to NK cells, dendritic cells, and a subset of T cells. The ability of HLA‐F to engage both activating and inhibitory receptors allows it to fine‐tune immune responses, contributing to both immune tolerance and activation depending on the context.

Unlike classical HLA‐I molecules, ER exit of HLA‐F is peptide‐ and TAP‐independent [192], instead relying on specific cytoplasmic motifs [197]. Due to its peptide‐independent expression and the relative stability of its open conformers, surface HLA‐F exists primarily in a peptide‐free state [186, 192, 196]. However, HLA‐F can also present peptides [186], though the mechanism of HLA‐F peptide presentation remains unclear, and no TCR‐mediated recognition of HLA‐F has been demonstrated to date. Peptide binding further fine‐tunes the immune regulatory functions of HLA‐F, as its peptide‐free and peptide‐bound forms engage distinct sets of inhibitory and activating NK receptors [102, 198].

HLA‐F possesses unique structural features in its peptide‐binding groove, where five of the 10 conserved residues essential for maintaining the classical HLA‐I binding groove architecture are substituted [198]. Through these alterations, the peptide‐binding groove adopts an open architecture that allows for the binding of long peptides [186], similar to HLA‐II molecules. The key residues crucial for shaping its unique peptide binding groove structure are highly conserved across primate species [199]. In addition, peptides eluted from HLA‐F are enriched in posttranscription modification [186], suggesting evolutionary adaptation of its binding groove for presenting modified peptides. These unusual properties of HLA‐F's peptide repertoire may play a crucial role in receptor recognition, warranting further investigation to elucidate their functional significance.

HLA‐F exhibits remarkably low polymorphism, with HLA‐F*01:01 present in around 90% of individuals [200, 201], reflecting its evolutionary adaptation as a universal immune modulator. This minimal genetic diversity, conserved across primate species [199, 202], is critical for maintaining HLA‐F's unique peptide‐receptive open conformation, preserving interactions with β2m‐free MHC‐I heavy chains and shaping its unique peptide repertoire [198]. These conserved structural features enable HLA‐F to function as a stable ligand for invariant immune receptors across diverse populations. Beyond ensuring consistent immune regulation, this minimal variation also reduces the risk of autoimmune dysregulation, highlighting its specialized role in maintaining immune tolerance and homeostasis.

### 4.2 HLA‐F Is a Possible Target for Next‐Generation Immunotherapies

In tumors, malignant cells frequently upregulate HLA‐F surface expression as an immune evasion strategy, engaging inhibitory receptors to suppress cytotoxic responses, which correlates with poorer clinical outcomes [203, 204, 205]. This positions HLA‐F as a critical modulator of the tumor microenvironment (TME). HLA‐F upregulation has also been reported during viral infections, including HCV [206], Japanese encephalitis virus [207], human adenovirus [208], BK polyomavirus [209], and HIV [102, 200]. During the infection, HLA‐F demonstrates protective functions by interacting with the activating NK receptor KIR3DS1 to stimulate antiviral immune responses. Similar to HLA‐E, antibody‐based strategies targeting HLA‐F may modulate the activation state of NK cells and T cells, with preclinical studies showing efficacy in suppressing tumor growth [210] (Table 2).

Although clinical immunotherapies targeting HLA‐F are lacking, emerging evidence suggests that HLA‐F may offer distinct advantages as an attractive target for immunotherapy. Unlike classical MHC‐Ia molecules that are frequently downregulated in cancers and infections, HLA‐F is often upregulated in these pathological contexts, ensuring target availability for therapeutic intervention. Its low polymorphism and predominant peptide‐receptive conformation simplify therapeutic development and enhance translatability across diverse populations [192, 196, 200, 201]. Importantly, HLA‐F's selective expression patterns minimize unintended damage to healthy tissues [192, 193, 194, 195]. Furthermore, HLA‐F may orchestrate broad immune modulation by engaging activating or inhibitory receptors on effector cells and regulating both innate and adaptive immune responses, offering opportunities for systemic immune network reprogramming [186]. In terms of adaptive immunity, HLA‐F not only associates with other HLA‐I molecules to regulate their surface half‐life [190], but can also be modulated to support cross‐presentation of classical HLA‐Ia molecules [191], which could be harnessed to amplify cancer vaccine responses or incorporated into CAR‐T or other ACT.

While these observations highlight HLA‐F as an emerging immunotherapeutic target in oncology and infectious diseases, significant gaps remain regarding its structural features, cell biology, and immune functions. It would be wise to understand these in more detail before trying to develop HLA‐F‐based therapies. The structural heterogeneity of HLA‐F and its capacity to deliver both activating and inhibitory signals complicate therapeutic targeting and raise safety concerns. As open conformers are the predominant form of HLA‐F expressed on the cell surface, current therapeutic strategies have focused on developing conformation‐specific antibodies rather than ligand‐specific reagents [102, 103]. However, a mono‐specific HLA‐F antibody is still lacking, let alone conformation‐specific ones [211], which is a major obstacle for both mechanical studies and clinical development of HLA‐F‐targeting therapies. In addition, as HLA‐F surface expression is largely peptide independent, it is challenging to modulate its surface level directly. HLA‐F is often expressed as a transient open conformer on the cell surface, complicating stable engagement by conventional biologics. Furthermore, while the highly selective expression pattern of HLA‐F may reduce the risk of off‐target immune activation, it also restricts the range of the diseases to which HLA‐F‐targeting therapies might apply. Overcoming these barriers will likely require alternative strategies, including cell‐permeable small molecules, targeted delivery platforms, or cellular therapies that exploit context‐dependent expression and recognition mechanisms. Together, these challenges underscore the need for deeper mechanistic insight and more precise targeting strategies before HLA‐F can be reliably harnessed for immunotherapy.

## 5 HLA‐G

### 5.1 HLA‐G Is Evolutionarily Optimized for Immune Tolerance

HLA‐G is a nonclassical MHC‐I molecule with highly specialized immune‐regulatory properties (Figure 4). Unlike the ubiquitous expression of classical HLA‐I molecules, HLA‐G expression is highly restricted to specific immuno‐privileged sites in healthy tissues [212]. Predominantly found in the placenta for maternal–fetal immune tolerance [98], the nonpolymorphic HLA‐G appears to substitute for HLA‐A, ‐B, and ‐C to protect trophoblasts from immune attack by T cells and NK cells, through direct binding to the inhibitory receptors LILRB1 and LILRB2 and by providing a potent version of the VL9 peptide to interact with the inhibitory NKG2A receptor (Table 1). HLA‐G is also expressed in cornea [213], pancreatic islets [214], and erythroid precursors [215] to mediate local immune suppression. Additionally, HLA‐G is present in the thymus, where it contributes to T cell education and central tolerance [216]. However, the induction of HLA‐G represents a common immune evasion strategy across a variety of diseases, including cancers [217, 218, 219, 220], transplantations [221], and viral infections [221, 222].

HLA‐G exerts its immunomodulatory effects by interacting directly with inhibitory receptors, including KIR2DL4, LILRB1, and LILRB2 [223]. While these receptors show broad binding to HLA‐I molecules, their affinity for HLA‐G is the highest [223]. LILRB1 is found on both lymphoid and myeloid cells [224], LILRB2 is restricted to myeloid cells [225], and KIR2DL4 is present on NK cells and a subset of CD8+ T cells [226]. These different expression patterns enable precise and cell‐specific immune modulation of HLA‐G in both innate and adaptive immune responses. Also, the VL9 peptide VMAPRTLFL derived from the signal sequence of HLA‐G has the highest affinity of all VL9 peptides for HLA‐E and thus affects its interactions with NKG2A/C‐CD94 [227].

A defining characteristic of HLA‐G is its generation of seven distinct isoforms via alternative splicing, comprising four membrane‐bound and three soluble forms [228]. While membrane‐bound isoforms deliver inhibitory signals locally, soluble HLA‐G binds to the surface receptors on nonadjacent target cells and gets internalized into the endosomal compartments, enabling a more flexible, contact‐independent mode of immune regulation [229, 230]. This dual mode of action provides the host with layered immune modulation mechanisms fine‐tuned for different physiological contexts. Only HLA‐G1 and G5 possess all three heavy‐chain domains (α1, α2, α3), enabling them to associate with β2m and peptides to form heterotrimers, similar to classical MHC‐I molecules [231]. Most HLA‐G isoforms, excluding HLA‐G3, can form homodimers [232], which enhance their affinity for inhibitory receptors [233]. Moreover, different isoforms and structural conformations of HLA‐G exhibit distinct receptor‐binding preferences [232]. Therefore, alternative splicing of HLA‐G allows maximal functional versatility with minimal genetic variability, enabling it to mediate complex and effective immune modulation.

Unlike the highly polymorphic classical HLA‐Ia molecules, HLA‐G exhibits limited polymorphism [228], minimizing the risk of HLA‐G being recognized as foreign during maternal–fetal and transplant tolerance. It also enables HLA‐G to perform as a uniform ligand for consistent interaction with inhibitory immune receptors, which is crucial for stable immune suppression across diverse individuals. In addition, the restricted polymorphism, along with the structural features of its peptide‐binding groove, constrains the diversity of HLA‐G‐binding peptides [234, 235], resembling that of HLA‐E. This restricted peptide repertoire reduces the likelihood of immunogenic peptide presentation, preventing unintended T cell activation.

While classical MHC‐I molecules are rapidly transported to the cell surface to present peptides to CD8+ T cells, HLA‐G often shows delayed or partial transport to the cell surface and is preferentially retained in the ER, largely due to an efficient ER retrieval motif in its cytoplasmic tail [236]. This limited surface expression minimizes antigen presentation and reduces the chance of HLA‐G being recognized by cytotoxic T cells. In addition, HLA‐G is more stable on the cell surface compared with classical MHC‐I molecules [237], which is crucial for delivering consistent inhibitory signals for immune tolerance. HLA‐G can form homodimers that are not only structurally stable with prolonged surface retention but also bind inhibitory receptors with higher affinity, thereby enhancing immune inhibition [43]. Moreover, HLA‐G possesses a truncated cytoplasmic tail that does not contain the tyrosine‐based endocytosis motif typically conserved in HLA‐I molecules [236], contributing further to its prolonged surface stability. HLA‐G can also be incorporated into exosomes and other extracellular vesicles [238], extending its immunomodulatory effects systemically. Collectively, these unique structural features and trafficking behaviors enable HLA‐G to effectively perform specialized immune‐regulatory functions. This is likely to be of great importance in the trophoblast, protecting it from maternal immune attack [239, 240].

The immunoregulatory functions of HLA‐G and its orthologs appear to be highly conserved, reflecting strong evolutionary pressure to maintain immune tolerance in critical physiological contexts. In NHPs, such as Paan‐AG in olive baboon and Mamu‐AG in RMs, these orthologs exhibit placental expression and support maternal–fetal tolerance [241, 242, 243]. They share key traits with HLA‐G, including restricted polymorphism, alternative splicing into both membrane‐bound and soluble isoforms, and truncated cytoplasmic tails that regulate intracellular transport pathways [241, 243].

### 5.2 HLA‐G Is a Promising Target for Next‐Generation Immunotherapies

Given the immuno‐inhibitory role of HLA‐G, its expression is also induced in cancers, which contributes to tumor progression and is closely related to clinical outcome [244, 245, 246]. Similar to HLA‐E, HLA‐G and its receptors are often upregulated during viral infection for immune escape, including HIV [247, 248], HCMV [249], herpes simplex virus (HSV)‐1 [222], rabies lyssavirus [222], HCV [250], HBV [251, 252], human papillomavirus [253], and SARS‐CoV2 [241]. Therefore, HLA‐G is a promising therapeutic target. As HLA‐G exists both in membrane‐bound form as well as soluble forms, soluble HLA‐G is a potential biomarker for diagnosing various cancers and infections [254, 255, 256, 257].

Several features make HLA‐G a highly promising target for immunotherapy. While classical MHC‐Ia molecules are often downregulated in the contexts of infections or cancers, HLA‐G is often upregulated under these conditions, ensuring the effectiveness of HLA‐G‐targeting therapies. Its low genetic variability simplifies therapeutic design and increases the likelihood of effectiveness across diverse patient populations. The coexistence of membrane‐bound and soluble HLA‐G isoforms allows it to modulate immune responses through both direct cell‐to‐cell interactions and paracrine signaling pathways. Additionally, HLA‐G acts on a wide range of immune cells, allowing for multilevel modulation of immune networks when targeted therapeutically.

Current monoclonal antibodies targeting HLA‐G are insufficient for therapeutic use, as they fail to recognize all isoforms and often cross‐react with classical HLA‐I molecules [212, 223]. The development of isoform‐specific antibodies is particularly challenging for HLA‐G, as HLA‐G isoforms are generated via alternative splicing and share overlapping extracellular domains [228, 258]. Besides, it is difficult to distinguish membrane‐bound HLA‐G from soluble ones with identical extracellular domains, which raises the risk of off‐target toxicity. Moreover, the engagement of multiple inhibitory receptors by HLA‐G limits the effectiveness of single‐antibody blockade strategies. Emerging platforms, such as nanobodies, bispecific antibodies, and structure‐guided epitope targeting, may help overcome these barriers [212]. A combination of different HLA‐G antibodies will likely be necessary to comprehensively inhibit all HLA‐G isoforms and their receptor interactions. Alternative strategies, including small‐molecule inhibitors and RNA interference [259, 260, 261], are also under investigation. Given the frequent coupregulation of HLA‐G with other immune checkpoint molecules, such as PD‐L1 and CTLA‐4, the clinical potential of combining HLA‐G‐targeted strategies with other immune checkpoint inhibitors is currently under active investigation [212]. In addition, as HLA‐E and HLA‐G cooperate to inhibit NK cells and a subset of T cells, combinatorial therapies targeting both HLA‐E and HLA‐G may offer enhanced efficacy in overcoming immune suppression and restoring antitumor or antiviral immunity.

While most HLA‐G‐targeting therapies focused on the development of specific blocking antibodies (Table 2), their development is challenged by the limited understanding of the complex spatial interactions between HLA‐G and its ligands [212]. Furthermore, these neutralizing antibodies might not effectively block soluble HLA‐G isoforms. Cell‐based strategies targeting HLA‐G‐expressing abnormal cells might be an alternative approach. However, careful evaluation is required to determine whether such interventions could disrupt immune tolerance, especially at the maternal–fetal interface. In addition, while HLA‐G is theoretically a good target for combinational therapy with existing ICBs, the efficacy and safety of these methods require further investigation. Overall, therapeutic targeting of HLA‐G will require a deeper understanding of its isoform‐specific functions and receptor interactions, together with carefully designed strategies to maximize antitumor or antiviral immunity without compromising immune tolerance.

## 6 CD1

### 6.1 CD1 is Evolutionarily Optimized for Lipid Presentation

CD1 represents a group of nonclassical MHC‐I molecules, whose major role is involved in presenting lipid antigens to T cells [262] (Figure 4 and Table 1). While CD1 is structurally similar to MHC‐I molecules, consisting of three extracellular domains that associate with β2m, its antigen binding groove is deeper and more hydrophobic, specialized in presenting lipid antigens rather than peptide antigens [263]. CD1 displays limited polymorphism, comprising five isoforms, each with distinct trafficking patterns, structural features, and antigen specificities [264]. Group 1 CD1 isoforms (CD1a, CD1b, and CD1c) are expressed mostly on thymocytes and specialized APCs, where they help shape the repertoire of lipid‐reactive T cells during thymic development while limiting widespread lipid autoreactivity [265, 266]. In contrast, the Group 2 CD1d molecule is broadly expressed, as it mediates systemic immune regulation through NKT cells [267]. CD1e mainly stays intracellularly and does not directly present lipid antigens for T cell recognition [268, 269].

Following arrival at the cell surface, CD1 molecules undergo endocytosis and are transported into the endosomal network, where they acquire lipid antigens for presentation [270]. The postinternalization trafficking routes are primarily determined by the cytoplasmic tail sequences, which differ among CD1 isoforms [271]. CD1a is predominantly localized at the plasma membrane, where it can readily capture extracellular lipids, enabling rapid antigen presentation [272]. However, a minor subset traffics to early endosomes and then recycles back to the surface through a shallow endocytic pathway [273]. In contrast, CD1b transiently passes through early endosomes before accumulating in late endosomes and lysosomes, a process facilitated by the adaptor proteins AP‐2 and AP‐3 [274]. CD1c and CD1d primarily traffic through the early and late endosomal compartments through an AP‐2‐dependent mechanism [275]. Extracellular lipids bind to different lipid‐binding proteins that direct them to distinct endosomal compartments for antigen loading [276]. Therefore, the adaptation of CD1 isoforms to specialize in surveying different compartments broadens the spectrum of lipid antigens available for presentation.

In contrast to other CD1 molecules, CD1e exhibits an intracellular localization and does not function as a direct antigen‐presenting molecule [268]. Instead, it participates in the processing of complex antigens and facilitates lipid loading and exchange for other CD1 proteins [276, 277, 278]. In immature dendritic cells, CD1e accumulates in the Golgi apparatus and is subsequently transported to lysosomes upon DC maturation [268, 269]. This unique trafficking pattern of CD1e contributes an additional mechanism for antigen processing, thereby enhancing the overall capacity of the CD1 system to present lipid antigens.

Unlike the peptide binding groove of other MHC‐I molecules, the antigen binding groove of CD1 is enriched with hydrophobic residues that enable the incorporation of lipid antigens [279]. This structural adaptation reflects their specialized role in lipid antigen presentation. Furthermore, different CD1 isoforms have evolved slightly distinct groove architectures, which serve as a molecular ruler to accommodate different lipid classes [280], thereby expanding the repertoire of lipids that can be presented to the immune system. CD1b possesses a large antigen‐binding groove composed of four pockets (A′, F′, C′, and T′), allowing it to bind long lipids, with chain lengths reaching up to C80 [281]. While CD1a/c/d both contain two key pockets (A′ and F′) optimized for C36‐42 lipids [282, 283, 284], variation in pocket shape and interconnectedness enables isoform‐specific lipid loading. CD1e exhibits a wide binding groove that facilitates rapid lipid exchange [285]. Together, these isoform‐specific structural features of CD1's antigen‐binding groove enable efficient presentation of a wide range of lipid peptides in a well‐controlled way.

While the relatively open conformation of CD1a enables lipid loading at neutral pH on the cell surface [284], other CD1 isoforms require exposure to acidic endosomal environments that induce partial structural relaxation, facilitating lipid access to their binding clefts [286, 287]. CD1e displays notable stability under acidic conditions, a property that might be crucial for its role in promoting lipid exchange or editing [288]. While the precise mechanism remains uncertain, CD1e likely functions similarly to HLA‐DM [278], which facilitates HLA‐II peptide loading within endosomes in a pH‐dependent manner, though HLA‐DM does not bind to ligands. In addition, acidic environments activate various acid‐dependent proteases, such as cathepsins, which cleave prosaposin to generate active lipid transfer proteins [289, 290]. Together, these pH‐dependent strategies allow for lipid antigens to be presented with temporal and spatial precision.

CD1 genes are universally present in all mammalian species, though isoform composition varies across species, indicating their essential role in immune function and evolutionary conservation under selective pressure [291]. Among primates, Group 1 CD1 proteins are well conserved, as rhesus homologs preserve key residues and motifs and can be recognized by corresponding human CD1 isoform‐specific antibodies [292, 293]. Besides, rhesus CD1 can also present pathogen‐derived lipids for recognition of human CD1‐restricted T cells. These findings highlight the crucial role of CD1‐mediated lipid antigen presentation in immune defense and validate NHP models as physiologically relevant systems for studying CD1 biology and developing clinical applications.

### 6.2 CD1 is a Promising Target for Next‐Generation Immunotherapies

CD1d‐restricted NKT cells provide defense against diverse bacterial pathogens, including *Streptococcus pneumoniae* [294, 295], *Borrelia burgdorferi* [296, 297], *S.typhi* [298], and *Sphingomonas capsulata* [299]. They also contribute to controlling viral infections, such as EBV [300], influenza A virus [301], HSV [302], CMV [303], HBV [304], respiratory syncytical virus [305], and SIV [306]. Beyond infectious diseases, NKT cells exhibit potent antitumor activity [307, 308, 309]. Given their broad‐spectrum antimicrobial and antitumor effects, CD1d‐restricted NKT cells, despite constituting a minor leukocyte population, represent promising therapeutic targets, particularly for vaccine development.

Functional studies on Group 1 CD1 molecules remain limited compared with CD1d due to their absence in laboratory mice, despite the development of the transgenic mouse model [310]. Their protective role is best characterized in Mtb infection, where CD1a–c present mycobacterial antigens to mediate protective immune responses [310, 311, 312, 313]. Emerging evidence suggests these molecules also contribute to defense against Staphylococcus infection [314] and tumor surveillance [315], though their roles in other pathogens require further investigation.

CD1 molecules present several compelling advantages that make them attractive targets for therapeutic development. First, unlike classical MHC molecules, CD1 exhibits minimal polymorphism and presents highly conserved lipid antigens [264], making it a promising candidate for universal vaccine development across genetically diverse populations. The distinct structural and functional features of CD1 isoforms enable broad recognition of pathogen‐derived lipid antigens [267, 270, 280], thereby expanding the landscape of targetable antigens beyond peptide‐based immune surveillance. Unlike peptide antigens, lipid antigens bypass complex processing requirements, allowing for more rapid and direct antigen presentation [272]. Moreover, CD1 surface expression is frequently upregulated in response to infection [316, 317], enhancing target availability for immune recognition, though certain viruses have evolved mechanisms to downregulate CD1 expression and evade immune detection [318, 319, 320, 321].

Beyond infectious diseases, CD1 also presents self‐lipids enriched in certain cancers, including leukemia [315], multiple myeloma [322], and ovarian cancer [323], with CD1‐restricted T cells demonstrating protective antitumor effects. This highlights the potential of CD1‐based approaches in cancer immunotherapy, especially in contexts where immunogenic peptide antigens are limited. The dual functionality of CD1‐restricted immunity is particularly valuable: Group 1 CD1 elicits adaptive T cell responses similar to MHC‐mediated immunity [310], while CD1d‐restricted iNKT cells bridge innate and adaptive immune defenses [324]. In cancer, the NKT cells engage CD1d in two major contexts. First, NKT cells can directly recognize and kill CD1d‐expressing tumor cells [322]. Second, CD1d is also expressed on immunosuppressive cells, such as tumor‐associated macrophages (TAMs) and myeloid‐derived suppressor cells [309, 325], which enables NKT cells to recognize these populations to remodel the TME. These properties position CD1‐targeted therapies as versatile immunological tools capable of eliciting broad and effective immune responses against a wide array of infectious and malignant diseases.

NKT cells are also closely linked to the pathogenesis of autoimmune diseases, exhibiting both immunoregulatory and proinflammatory functions. Their involvement has been reported in systemic lupus erythematosus (SLE), rheumatoid arthritis (RA), autoimmune hepatitis (AIH), multiple sclerosis (MS), Type 1 diabetes (T1D), and experimental autoimmune encephalomyelitis [326, 327]. Preclinical studies exploring the therapeutic potential of NKT cells in autoimmune diseases have yielded promising results [328], though further research into the immunoregulatory mechanisms of NKT cells and the translational potential of NKT‐based therapies from murine models to human diseases is required.

In addition, CD1a has a distinctive role in cutaneous and barrier immunity. Expressed abundantly on Langerhans cells [329], CD1a presents self and foreign lipid antigens within the skin microenvironment [330], making it uniquely positioned to sense barrier perturbations and essential for host‐pathogen defenses [331]. CD1a‐reactive T cells are enriched in the skin, which can produce a diverse range of cytokines, contributing to host defenses as well as cutaneous inflammation under certain circumstances [329, 330, 332, 333]. Lipid‐modifying enzymes, especially phospholipase A2, generate a range of lipid antigens, which are essential for the activation and expansion of CD1a‐autoreactive T cells and are hallmarks of chronic inflammation [334]. These findings underscore the potential of CD1a as a critical therapeutic target for a broad range of inflammatory skin diseases.

Given the specialized antigen repertoire of CD1 molecules, administration of lipid antigens offers a direct approach to selectively modulate CD1‐restricted immune responses. Activation of iNKT cells using α‐galactosylceramide (α‐GalCer) and its analogs has shown efficacy in suppressing solid tumor growth and controlling chronic viral infections in preclinical models [335, 336, 337, 338, 339], and several clinical trials are currently underway to evaluate the therapeutic potential of these agonists in patients (Table 2). The deep hydrophobic antigen‐binding groove of CD1d has been actively exploited to design synthetic lipid ligands with tailored immunological effects [338, 340]. Structure–activity relationship studies of α‐GalCer have enabled precise modification of lipid tails and head groups to better complement the CD1d groove, yielding derivatives with enhanced binding stability and biased cytokine production [341]. However, while such cargo‐based strategies allow for precise immune tuning, it is challenging in practice due to the complexity and relatively limited understanding of lipid metabolism and trafficking. Indirect strategies aimed at modulating lipid biosynthesis, processing, or intracellular trafficking pathways may also reshape the CD1 antigen repertoire, but these approaches may cause broad and unintended metabolic effects.

Beyond antigen manipulation, CD1‐expressing cells themselves may be targeted using monoclonal antibodies or engineered cell‐based therapies [323, 342, 343, 344, 345], which have demonstrated encouraging results in preclinical studies, particularly in enhancing antitumor immunity and combating intracellular pathogens. Nevertheless, the broad expression of CD1 molecules raises concerns about disrupting immune homeostasis, and the development of lipid antigen‐specific antibodies remains technically challenging. Overall, while CD1 molecules offer a promising platform for immunotherapy through cargo manipulation, receptor engagement, and cellular targeting, the inherent complexity of lipid antigen biology and the risk of perturbing immune equilibrium remain major challenges that must be addressed to enable safe and effective clinical translation.

## 7 MR1

### 7.1 MR1 is Evolutionarily Conserved for the Presentation of Metabolite‐Derived Ligands

The MHC‐I‐related molecule MR1 is specialized in presenting microbial riboflavin (vitamin B) metabolites and related small molecules to MAIT cells [346, 347] (Figure 4). In contrast to MAIT cells, a less abundant subset of MR1‐restricted T (MR1T) cells recognizes self‐antigens in an MR1‐dependent manner [348] (Table 1). Unlike highly polymorphic classical MHC molecules, MR1 is nearly monomorphic across human populations, underscoring its evolutionary conserved role in microbial immune surveillance [349]. Although the MR1 protein is ubiquitously expressed across most cell types, it is predominantly retained intracellularly, and its surface expression is tightly regulated by ligand binding [350]. Thus, MR1 is particularly enriched at mucosal surfaces, where it captures riboflavin biosynthetic intermediates derived from bacteria and fungi [351, 352].

Structurally, the antigen binding groove of MR1 is narrow and hydrophobic, partially occluded by bulky aromatic residues, making it well suited to accommodate small‐molecule ligands [353]. The A′ pocket is enriched in basic residues that facilitate Schiff base formation with the ligand, a covalent interaction that stabilizes MR1, promotes proper folding, and enables translocation to the cell surface [354]. Furthermore, this binding cavity exhibits conformational plasticity, allowing its binding to a structurally diverse range of ligands [353]. These unique structural characteristics collectively enable MR1 to function as a specialized sensor of microbial‐derived metabolites and an efficient presenter of nonpeptide antigens to MAIT cells.

MR1 undergoes spontaneous alternative splicing, generating multiple isoforms with distinct structural and functional properties [351]. While most studies to date have focused on the full‐length MR1A isoform, the functions of other truncated versions lacking the α3 domain are not well characterized [355]. It was reported that MR1B could present ligands at the plasma membrane and activate MAIT cells in vitro, functioning as homodimers independent of β2m association [351]. MR1B can also modulate the strength of MAIT cell responses elicited by MR1A [356, 357]. MR1C is predicted to be soluble, though its function remains poorly defined [357]. These findings suggest that MR1 isoforms possess distinct features and may work in concert to precisely regulate MAIT cell activity in a manner reminiscent of HLA‐G [229].

The intracellular trafficking dynamics of MR1 resemble those of MHC‐E. MR1 surface expression is low due to ER retention and intrinsic surface instability [356, 358, 359], which is largely attributed to the limited availability of MR1 ligands [360]. Upon ligand binding, either through exogenous addition or microbial infection, MR1 exhibits increased surface stability and expression [123, 347, 356]. Unlike classical MHC‐I molecules, the α3 domain of MR1 only interacts weakly with β2m [361], and ligand loading is a prerequisite for effective refolding and β2m association [356]. Additionally, MR1 possesses a tyrosine‐based internalization motif, which can be recognized by AP‐2, contributing to its rapid turnover via clathrin‐mediated endocytosis [362]. These mechanisms ensure that only ligand‐loaded MR1 reaches the surface, allowing microbial vitamin B‐derived antigens to be presented in a timely and precisely regulated manner while minimizing the risk of unintended MAIT cell activation.

While the ER serves as the primary site of MR1 antigen loading, additional pathways have been identified [363, 364]. MR1 molecules can undergo ligand exchange within endosomal compartments, and those that acquire ligands through endocytosis, phagocytosis, or autophagy can subsequently be transported back to the cell surface [363]. This process may be similar to MHC‐II molecules, as CD74 and HLA‐DM have been shown to interact with MR1 and modulate its endosomal routing and ligand exchange, thereby influencing MAIT cell activation [365]. In some cases, soluble ligands can bind MR1 directly at the plasma membrane, either by displacing prebound ligands or engaging a minor pool of ligand‐free MR1 molecules [356, 366], though this appears to be a relatively inefficient mechanism. These alternative antigen‐loading routes expand the range of MR1‐presented ligands and potentially reduce the chances of microbial immune evasion.

MR1 is one of the most evolutionarily conserved members of the MHC family, with the human and mouse MR1 proteins sharing approximately 90% sequence homology within the α1 and α2 domains [367]. This high degree of conservation, particularly within the antigen‐presenting interface, reflects the essential and invariant nature of MR1's immunological functions. Indeed, both mouse and human MAIT cells can recognize MR1 molecules across species [368], demonstrating significant cross‐reactivity with mammalian MR1 orthologs. Moreover, alternative splicing of MR1 has been observed in NHPs and other mammal species [357, 369], indicating that the structural and regulatory features of MR1 are preserved throughout evolution. The strong evolutionary conservation of MR1 suggests that it has evolved under purifying selection, likely due to its specialized role in recognizing a narrow but critical set of microbial‐derived vitamin B metabolites that are indispensable for microbial survival.

### 7.2 MR1 is a Promising Target for Next‐Generation Immunotherapies

The MR1–MAIT axis offers several unique advantages over the conventional MHC‐I–CD8+ T cell pathway in immunotherapy. Unlike classical MHC‐I molecules, MR1 is monomorphic [349], making MR1‐targeted therapies universally applicable across the human population. This broad compatibility simplifies therapy design, lowers manufacturing cost, and enhances translational potential. Furthermore, MR1 is highly conserved across mammalian species [357, 367, 368, 369], facilitating preclinical validation in multiple animal models. Under steady‐state conditions, MR1 is predominantly retained in intracellular compartments [356, 358, 359], forming a reservoir that can be rapidly mobilized to the cell surface upon microbial infection. This tightly regulated trafficking minimizes off‐target activation while enabling rapid antigen presentation in response to infection.

MAIT cells are prearmed with proinflammatory cytotoxic granules and cytokines, and upon activation, they markedly upregulate cytotoxic granule components, enabling an immediate effector response [370]. They are also enriched at mucosal surfaces [351, 352], positioning them at the frontline of host defense. Moreover, MAIT cells express several NK‐activating receptors [371], suggesting that they may exert rapid cytotoxic effects through TCR‐independent pathways, potentially extending their activity to tumors with low or absent MR1 expression. These features collectively support rapid and localized immune responses within hours of microbial encounter—an advantage over conventional CD8+ T cells, which require clonal expansion and differentiation.

A key immunological advantage of MAIT cells lies in their unique recognition of microbial riboflavin metabolites, which are evolutionarily conserved among a wide range of bacteria and fungi. This reduces the risk of immune escape and allows for broad‐spectrum targeting of diverse pathogens using a single ligand‐based approach. Moreover, since these microbial ligands are absent in host cells, the risk of off‐target cytotoxicity or autoimmunity is low. Unlike peptide antigens that require complex intracellular processing and MHC‐I loading, MR1 directly presents small‐molecule ligands, enabling faster and more efficient antigen presentation. From a therapeutic perspective, these ligands are generally easier to synthesize and modify than peptides, though further research is needed to identify potent and stable MR1 ligands optimized for clinical application [372] (Table 2).

MR1 presents riboflavin metabolite derivatives to MAIT cells, enabling the recognition and immune control of a broad range of bacterial pathogens. In murine models, MR1‐restricted MAIT cells play a crucial role in controlling infections such as Mtb, Francisella tularensis, and Klebsiella pneumoniae, with MR1 deficiency resulting in impaired bacterial clearance and increased susceptibility to infection [373, 374]. In humans, MAIT cells are activated during infections with Mtb, Shigella dysenteriae, and Vibrio cholerae, and their depletion or functional impairment has been associated with more severe disease outcomes and compromised mucosal immunity [373, 375, 376]. These findings collectively underscore the critical role of MR1 in shaping early antibacterial immune responses through MAIT cell activation.

Recent studies have also highlighted the important role of MAIT cells in cancer, though this area remains largely unexplored [377, 378]. Similar to NKT cells, MAIT cells can be engineered to target TAMs and modulate the immunosuppressive TME [379, 380]. However, MAIT cells have also been reported to exhibit tumor‐promoting functions in some contexts [381], indicating that their role in cancer is complex and requires further investigation. Besides, MAIT cells also contribute to various autoimmune diseases, including SLE, MS, T1D, AIH, RA, inflammatory bowel disease, ulcerative colitis, and autoimmune liver diseases [382, 383, 384]. Given the limited understanding of the precise roles of MAIT in these diseases and that the results of some studies are conflicting, more research into their functions is required for further exploration of potential translational values [385, 386].

Peripheral blood MAIT cell levels could serve as biomarkers for disease progression and immune reconstitution, as they decline in active TB patients but rebound following therapy [387]. Moreover, MR1 ligands can act as adjuvants to enhance immune responses against bacterial infection [388]. Notably, this adjuvant function could be extended to viral vaccines, though viruses are devoid of the riboflavin biosynthesis machinery [389]. Early activation of MAIT cells is crucial for eliciting effective adaptive immune responses following antiviral vaccination [390, 391]. Apart from MAIT cells, MR1T cells can recognize self‐antigens presented by tumor cells. Recently, MR1T cells have shown promise in pan‐cancer recognition [392, 393], though this remains disputed and requires further validation [394]. Engineered MR1T cells exhibit potent tumor‐killing activity in vitro, capable of recognizing MR1‐expressing cancers across diverse donors [395]. These findings highlight the therapeutic potential of MR1T cells as a universal strategy for cancer immunotherapy.

The unique metabolite antigen repertoire of MR1 makes cargo‐based strategies especially attractive. As MR1 surface expression requires ligand loading, direct administration of MR1 agonists could modulate MR1 surface level and potentially activate MAIT cells or other MR1‐restricted T cells. Although small‐molecule ligands are generally cheaper and easier to manufacture than peptide ligands, their therapeutic application to MR1, which loads peptides intracellularly, requires efficient cell permeability and precise access to intracellular compartments while minimizing off‐target effects and potential toxicity [372]. Monoclonal antibodies are conceptually appealing, but it is technically challenging to generate high‐affinity antibodies that specifically recognize MR1 loaded with tumor/pathogen‐specific ligands. MR1‐restricted T cells raise the possibility of a universal antitumor therapy, since MR1 is almost monomorphic across humans and some MR1T clones have broad tumor recognition [392, 393]. However, this promise is currently limited by the incomplete understanding of the tumor‐associated MR1 ligands. Therefore, successful clinical translation of MR1‐targeted therapies will require systematic identification and characterization of tumor‐derived MR1 ligands as well as the development and safety profiling of MR1‐targeted reagents.

## 8 Conclusions and Future Perspectives

**FIGURE 5:**
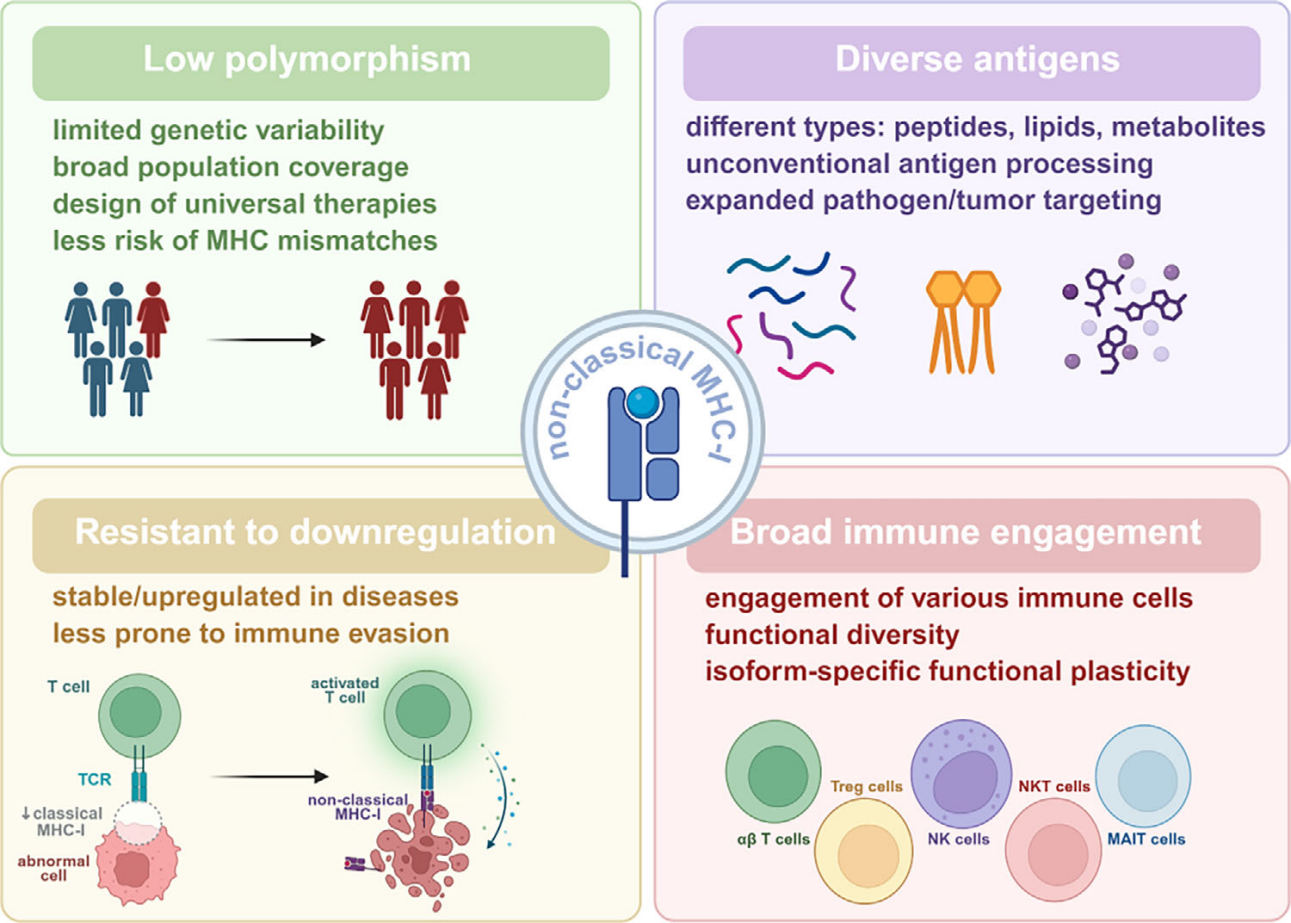
Advantages of nonclassical MHC‐I molecules for next‐generation immunotherapy. Nonclassical MHC‐I molecules exhibit several features that make them attractive for innovative immunotherapeutic strategies. Compared with classical MHC‐I molecules, they show low genetic polymorphism, enabling broader population coverage, facilitating universal therapy design, and reducing the risk of alloreactivity. They present diverse antigen types, including peptides, lipids, and metabolites, and adopt unconventional pathways, expanding the scope of pathogen and tumor targeting. Their expression is often stable or upregulated in disease settings, making them less susceptible to immune evasion via MHC downregulation. Furthermore, nonclassical MHC‐I molecules engage a wide range of immune cells, offering functional plasticity and novel avenues for fine‐tuned immune modulation.

Nonclassical MHC‐I molecules represent a promising frontier in immunotherapy, offering distinct advantages over their classical counterparts (Figure 5). These molecules exhibit limited polymorphism, unique trafficking behaviors, specialized antigen‐presentation capabilities, and dual engagement of innate and adaptive immune cells. These features position them as versatile targets for overcoming the critical limitations of current therapies dependent on classical MHC‐I molecules, particularly MHC‐I downregulation in malignancies and the restricted population coverage imposed by MHC polymorphism. From unconventional antigen presentation to precise immune modulation, nonclassical MHC‐I molecules provide novel therapeutic opportunities against cancers, infections, and immune‐mediated diseases.

HLA‐E is clinically tractable with monoclonal antibodies that block the HLA‐E–NKG2A axis (e.g., monalizumab), and early‐phase clinical trials have shown the efficacy of this strategy across several tumors [396, 397] (Table 2). In addition, vaccines designed to elicit HLA‐E‐restricted CD8+ T cell responses represent another promising avenue. Although human vaccine trials remain at an early stage, strong preclinical evidence demonstrates that induction of MHC‐E‐restricted CD8+ T cells offers robust protection in NHP models [157, 159, 160]. The biology and translational potential of HLA‐F remain largely unexplored, but emerging studies suggest that antibody‐based approaches targeting the HLA‐F–KIR3DS1 axis are a promising therapeutic strategy [208]. While numerous HLA‐G‐targeting therapies have been proposed, antibodies directed against HLA‐G or its inhibitory receptor LILRB1, either as monotherapies or in combination with existing ICBs, are currently the most actively investigated approaches in clinical development [212]. In parallel, HLA‐G has been established as a clinically relevant biomarker with predictive value for therapeutic response and cancer prognosis [244, 245, 246]. CD1 is most tractable via cellular therapies that exploit its unique capability to present lipid antigens to NKT cells. Cargo‐based approaches and iNKT‐based strategies have shown encouraging results in clinical studies [335, 336, 337, 338, 339, 398]. Small‐molecule ligands are currently the most attractive MR1‐targeting strategies, as such small‐molecule ligands are typically cheaper and simpler to produce than peptide ligands [372].

Beyond their potential as stand‐alone treatments, therapies targeting nonclassical MHC‐I molecules offer opportunities to complement and enhance existing immunotherapeutic strategies. Nonclassical MHC‐I pathways, such as HLA‐E/NKG2A and HLA‐G/LILRB1, operate in parallel with classical immune checkpoints and are frequently coupregulated with classical immune checkpoints, like PD‐L1, in tumor cells [93, 399, 400], creating complementary inhibitory networks that suppress both NK and T cell function. Consequently, blockade of a single checkpoint may be insufficient to fully restore the antitumor immunity, providing a strong rationale for combinatorial immune checkpoint inhibition strategies [181, 401]. However, simultaneous targeting of multiple inhibitory axes may increase the risk of autoimmunity, so the potential adverse effects should be carefully examined. At the same time, antagonistic interactions must be considered. For example, vaccines that elicit robust HLA‐E‐restricted T cell responses may upregulate HLA‐E surface expression and reinforce NK inhibition [121, 124, 175], diminishing the efficacy of NK‐cell‐based therapies.

Several challenges must be addressed to realize the clinical potential of nonclassical MHC‐I molecules. Many fundamental aspects of their biology remain incompletely understood, including ligand processing dynamics, receptor interaction networks, and context‐dependent regulation. Moreover, improving the delivery of therapeutic agents has emerged as a critical strategy to overcome these obstacles, and methods like hydrogels, nanoparticles, and biomaterial scaffolds are being actively developed to improve immunotherapy efficacy and reduce toxicity by enhancing targeted delivery and controlled release of immunotherapeutic agents [396, 402]. Future research should prioritize the discovery of novel ligands, structure–function analyses of receptor interactions, and the development of isoform‐specific modulators. Combinatorial therapies that integrate nonclassical MHC‐targeting approaches with established immunotherapies, such as ICB or CAR‐T cell therapy, may offer synergistic benefits and help overcome current therapeutic resistance. Collaborative efforts integrating fundamental immunology, computational biology, and clinical research will be critical to unlocking the full potential of these molecules and developing safer, broader, and more effective immunotherapies.

Although the limited polymorphism of nonclassical MHC‐I molecules represents a clear advantage, this alone does not guarantee that therapies targeting these molecules will be universally applicable. For HLA‐E, functional differences between the two common alleles (01:01 and 01:03) in surface stability and peptide repertoire may influence therapeutic efficacy [116, 403, 404, 405], particularly for strategies relying on HLA‐E–peptide complexes. HLA‐G and HLA‐F display context‐specific expression patterns and functional effects [195, 406]. CD1a–e are expressed in an isoform‐ and tissue‐dependent manner, and their levels can change during immune activation or inflammation [407]. MR1 surface expression is tightly regulated and inducible by specific ligands and inflammatory signals [354, 408].

Finally, while we have highlighted the advantages of targeting nonclassical MHC‐I molecules for immunotherapy, these benefits are accompanied by challenges that require careful consideration. The conserved nature of nonclassical MHC‐I molecules may impose strong and uniform selective pressure on pathogens or tumors, potentially facilitating the emergence of immune escape mechanisms that circumvent these pathways more rapidly than would occur for highly polymorphic classical MHC‐I targets. However, the absence of such T cell responses before initiating therapy could mean that there is no pre‐existing escape, offering an advantage, which could be enhanced by focusing on several epitopes at the same time. Furthermore, nonclassical MHC‐I molecules that function at the interface of innate and adaptive immunity, could enable coordinated advantageous modulation of both arms of the immune system. However, their modulation might also result in broad immune reprogramming, so thorough evaluation of potential side effects is required. Because nonclassical MHC‐I molecules engage multiple receptors and simultaneously influence different immune cell types (Table 1), therapeutic targeting could induce multilevel modulation that might be difficult to balance precisely. Moreover, as these molecules play key roles in maintaining immune homeostasis, particularly at mucosal sites where they are highly expressed, therapeutic interventions must be carefully balanced to elicit effective immune responses without disrupting immune tolerance. In addition, many strategies targeting nonclassical MHC‐I molecules also affect classical MHC‐I responses due to shared mechanisms such as antigen trimming and loading. All of these factors, if recognized should be manageable so that a precise and critical balance between classical and nonclassical MHC‐mediated immune responses will lead to the safe and effective clinical translation of these therapeutic approaches.

## Author Contributions

W.H. and A.J.M. conceived the review. W.H. drafted and wrote the manuscript. A.J.M. reviewed and edited the manuscript. All authors approved the final version of the manuscript.

## Ethics Statement

The authors have nothing to report.

## Conflicts of Interest

The authors declare no conflicts of interest.

## Data Availability

All data generated and/or analyzed during the current study are included in this published article.
